# Transcriptomic and physiological analyses reveal different grape varieties response to high temperature stress

**DOI:** 10.3389/fpls.2024.1313832

**Published:** 2024-03-08

**Authors:** Feifei Dou, Fesobi Olumide Phillip, Gang Liu, Jingjing Zhu, Lipeng Zhang, Yongkang Wang, Huaifeng Liu

**Affiliations:** Key Laboratory of Special Fruits and Vegetables Cultivation Physiology and Germplasm Resources Utilization of Xinjiang Production and Construction Crops, Agricultural College, Department of Horticulture, Shihezi University, Shihezi, China

**Keywords:** grape, high temperature stress, transcriptome, heat shock protein, plant physiology

## Abstract

High temperatures affect grape yield and quality. Grapes can develop thermotolerance under extreme temperature stress. However, little is known about the changes in transcription that occur because of high-temperature stress. The heat resistance indices and transcriptome data of five grape cultivars, ‘Xinyu’ (XY), ‘Miguang’ (MG), ‘Summer Black’ (XH), ‘Beihong’ (BH), and ‘Flame seedless’ (FL), were compared in this study to evaluate the similarities and differences between the regulatory genes and to understand the mechanisms of heat stress resistance differences. High temperatures caused varying degrees of damage in five grape cultivars, with substantial changes observed in gene expression patterns and enriched pathway responses between natural environmental conditions (35 °C ± 2 °C) and extreme high temperature stress (40 °C ± 2 °C). Genes belonging to the HSPs, HSFs, WRKYs, MYBs, and NACs transcription factor families, and those involved in auxin (IAA) signaling, abscisic acid (ABA) signaling, starch and sucrose pathways, and protein processing in the endoplasmic reticulum pathway, were found to be differentially regulated and may play important roles in the response of grape plants to high-temperature stress. In conclusion, the comparison of transcriptional changes among the five grape cultivars revealed a significant variability in the activation of key pathways that influence grape response to high temperatures. This enhances our understanding of the molecular mechanisms underlying grape response to high-temperature stress.

## 1 Introduction

Grapes have a long cultivation history and rich germplasm resources. According to latest data from the National Bureau of Statistics, in 2022, China’s grape production is 15.3779 million tons (http://data.stats.gov.cn). Similarly, fresh grapes accounted for approximately 42% of the total. The projected figures indicate that the fresh and dried grape planting area in Xinjiang will remain stable at approximately 0.1 million hectares by 2025. Furthermore, it is anticipated that the yield will exceed 2.5 million tons during this period (Wu et al., 2021). Extreme temperature events are predicted to occur more frequently, intensively, and for longer periods. In several regions, the noon temperature can reach 40°C or higher. High temperatures (HTs) adversely affect the development and composition of grapes, posing a significant threat to their yield and quality. This could undermine the environmental and economic sustainability of grape production (Schultz and Jones, 2010; Fraga et al., 2016). Previous studies on the response and adaptation of grapes to HT have primarily focused on morphological and physiological changes. These include changes in photosynthesis, hormone levels, and antioxidant systems (Wang and Li, 2006; Wang et al., 2009; Luo et al., 2011; Bita and Gerats, 2013). With the availability of the grape genome sequence, more studies have focused on transcriptomic and proteomic changes in response to heat stress. Several transcriptomic studies have explored the effects of heat stress on plant species, such as potatoes, tomatoes, rice, tobacco, and Arabidopsis (Higashi et al., 2015; González-Schain et al., 2016; Keller et al., 2018; Rahmati et al., 2018; Blair et al., 2019; Liu G. et al., 2020; Liu H. et al., 2020; Tang et al., 2020; Bineau et al., 2021; Liu et al., 2021).

Despite the importance of understanding thermotolerance and heat stress responses in grape leaves, evidence of the underlying molecular pathways is limited. However, the mechanism of grape response to extreme temperature stress remains unclear. Heat shock proteins (HSPs) and heat shock transcription factors (HSFs) play significant roles in thermotolerance. The accumulation of HSPs and expression of *HSFs* has been shown to be related to this process (Larkindale and Huang, 2004; Charng et al., 2006). The role of HSPs in scavenging reactive oxygen species (ROS), maintaining cell membrane integrity, and producing antioxidants and osmolytes is crucial for protecting plants from heat stress. Heat-responsive genes, in combination with transcription factors (TFs), are also induced in response to heat stress. Several transcription factor families, including DREB, MYB, NAC, HSF, and bZIP, play critical roles in heat stress response (Jiang et al., 2020). To better understand the mechanisms of the plant heat stress response, it is important to investigate the roles of different TFs involved in the process.

Transcriptomic analyses have provided comprehensive insights into the mechanisms underlying heat stress responses in grapes (Liu et al., 2012; Rienth et al., 2014; Jiang et al., 2017; Lecourieux et al., 2017; Kim et al., 2018). However, less attention has been paid to heat stress-related metabolic pathways and the molecular signaling networks involved in grapes. One promising avenue of research is examining endoplasmic reticulum (ER) protein processing in plant responses to heat stress (Li et al., 2015; Wang et al., 2017; Jin et al., 2020; Moreno, 2021). Many ER proteins such as HSPs and chaperones, are critical for ER function (Park and Seo, 2015). Accumulation of unfolded or misfolded proteins can lead to ER stress, which activates the unfolded protein response (UPR) to relieve stress. ER-associated degradation (ERAD) is a process that occurs in the ER and involves degradation of misfolded and unfolded proteins through a ubiquitin/proteasome mechanism. Although the above findings suggest a relationship between ERAD, UPR, and heat stress, the specific molecular mechanisms underlying this relationship have not been explored in grapes.

In this study, we selected five representative grape varieties (‘Xinyu,’ ‘Miguang,’ ‘Summer Black,’ ‘Beihong,’ and ‘Flame seedless’) with strong cultivation adaptability and wide planting area in Xinjiang as test materials. We compared the physiological, biochemical, and transcriptome data of five grape varieties after high-temperature treatment by simulating the natural high-temperature environment in a solar greenhouse. The objective of this study was to determine the heat tolerance levels of different grape varieties and identify important genes that may be related to HT resistance, including HSPs and TFs. Additionally, we detected changes in metabolic pathways after exposure to HT stress. This study contributes to a better understanding of transcriptome defense mechanisms associated with heat tolerance in grapes.

## 2 Materials and methods

### 2.1 Experimental materials, high-temperature treatment, and sample collection

This experiment was conducted at the Shihezi University Experiment Station greenhouse. The experimental materials consisted of 2-year-old seedlings of five grape varieties—’Xinyu’ (XY *Vitis vinifera L.*, Originating from Xinjiang, China), ‘Miguang’ (MG *V. vinifera L. × Vitis labrusca*, originating from Hebei, China), ‘Summer Black’ (XH *V. vinifera × V. labrusca*, Origin in Japan), ‘Beihong’ (BH *V. vinifera × V. amurensis*), and ‘Flame seedless’ (FL *V. vinifera L*, Origin in the United States)—grown in cultivation bags measuring 27 cm (height) × 30 cm (diameter), with a culture medium of pastoral soil and organic matter in a ratio of 2:1. The plant row spacing was set at 80 cm × 100 cm, and soil moisture was maintained between 31% and 35%. Consistent field management practices were implemented for all plants throughout the experiment.

**Figure 1:**
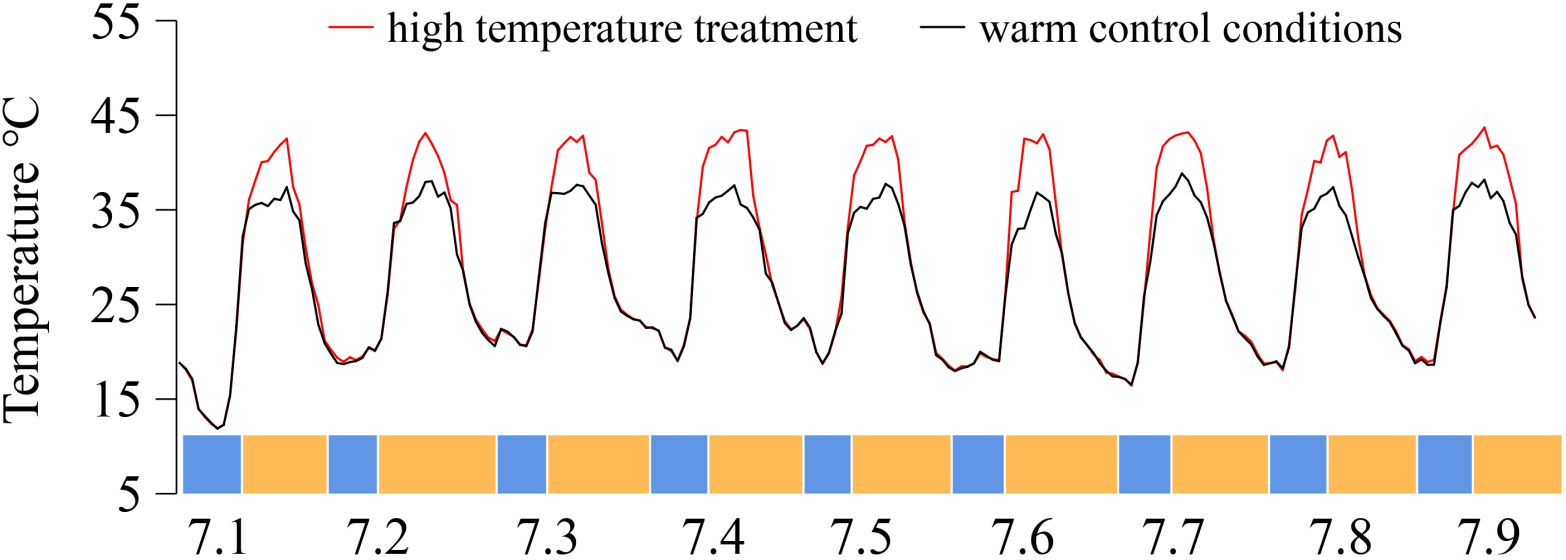
Temperature was recorded of the greenhouse from 1–9 July 2019, China. On the X-axis, blue represents night temperatures, whereas yellow denotes day temperatures.

Ten plants with strong growth were randomly selected from each grape cultivar for temperature treatment. The temperature in the solar greenhouse was regulated by an exhaust fan, monitored by a temperature sensor, and maintained at 40 °C ± 2 °C (T: high-temperature treatment). If the temperature rose above 40 °C, the exhaust fan started to work, and the temperature was reduced. The high-temperature period was from 12:00 to 19:30 each day, whereas routine management was conducted during the other periods. The control temperature was set at 35 °C ± 2 °C (CK: control treatment, natural solar greenhouse temperature). In this experiment, a MicroLite-U disk temperature recorder was used to record the temperature once every hour for continuous monitoring of the greenhouse temperature. The temperature data are shown in **Figure 1**.

We conducted high-temperature (HT) treatment from 1 to 7 July 2019 and collected grape leaves on the 7th day of HT treatment. At the same time, the leaves of grapes cultured at 35°C were collected as controls. Each grape variety randomly selected robust young leaves near the functional leaves of sections 9–11 from bottom to top and wrapped in tin foil. They were immediately frozen in liquid nitrogen and stored at −80°C for RNA seq analysis.

### 2.2 Physiological measurements

Four physiological indexes were measured for each grape cultivar to analyze the physiological changes induced by heat stress. Superoxide dismutase (SOD) activity was determined by the nitrogen blue tetrazolium method, peroxidase (POD) activity was determined using the guaiacol method, and catalase (CAT) activity was detected by ultraviolet spectrophotometry (Cheng et al., 2018). The method described by Liu et al. (2013) was used to determine the production rate of O^2−^. The malondialdehyde (MDA) and hydrogen peroxide (H_2_O_2_) contents were determined using the method described by Li et al. (2015). The method described by Lichtenthaler (1087) was used to determine chlorophylla (Chla) and chlorophyllb (Chlb). A fluorometer (FMS-2, Hansatech, UK) was used to measure chlorophyll fluorescence. The photosynthetic parameters were investigated using a photosynthesizer (Li-6800, LI-COR, US).

### 2.3 RNA-Seq preparation and data analysis

On the 7th day of high-temperature stress at 4 p.m., each grape variety randomly selected robust young leaves near the functional leaves of sections 9–11 from bottom to top, immediately frozen with liquid nitrogen, and stored at −80°C for RNA-seq analysis. Total RNA from 30 samples was extracted using TRIzol Reagent (Invitrogen, Canada). The total RNA was quantified using a 2100 Bioanalyzer (Agilent Technologies). These libraries were then sequenced by Shanghai Personal Biotechnology Cp. Ltd. using Next-Generation Sequencing (NGS) technology based on the Illumina sequencing platformAfter on-line sequencing, the samples generated raw Data of FASTQ and were statistically calculated We used the DESeq R package to analyze differences in gene expression (DEGs) and screened for DEGs that met the following conditions: | log2foldchange | ≥1 and p-value ≤0.05.

### 2.4 Validation of gene expression by qRT-PCR

QRT-PCR was performed on nine DEGs related to HT treatment, repeated three times for each sample. QPCR for the relative expression levels of target genes was performed using the CFX Connect Real-time PCR Detection System (BioRad, USA) and SYBR® Premix Ex TaqTM II (TaKaRa). Total RNA was extracted from the grapes using the TRIzol reagent (Invitrogen). This kit was used for gDNA removal (TransGen Biotech, Beijing, China). Actin was used as an internal reference control for data normalization. The expression level of each target gene was calculated using the 2^−△△^Ct method (Ma et al., 2015).

### 2.5 WGCNA and correlation analyses of physiological index-related genes

We built gene co-expression networks based on the DEGs using weighted correlation network analysis (WGCNA) in the R package (Langfelder and Horvath, 2008). DEGs from the five grape varieties (high-temperature and control-treated samples) were used for the co-expression analysis. All physiological indices were examined for correlation with the modules and all genes in each module. Significant high-temperature tolerance-related modules were detected based on the highest correlation values with the physiological indices.

### 2.6 Statistical analysis

SPSS software (version 19.0) was used for the analysis of variance (P ≤0.05), and Duncan’s method was used for multiple comparisons and significance tests. The results are presented as mean ± standard error of three replicates.

## 3 Result

### 3.1 Physiological changes in five grape varieties under HT

**Figure 2:**
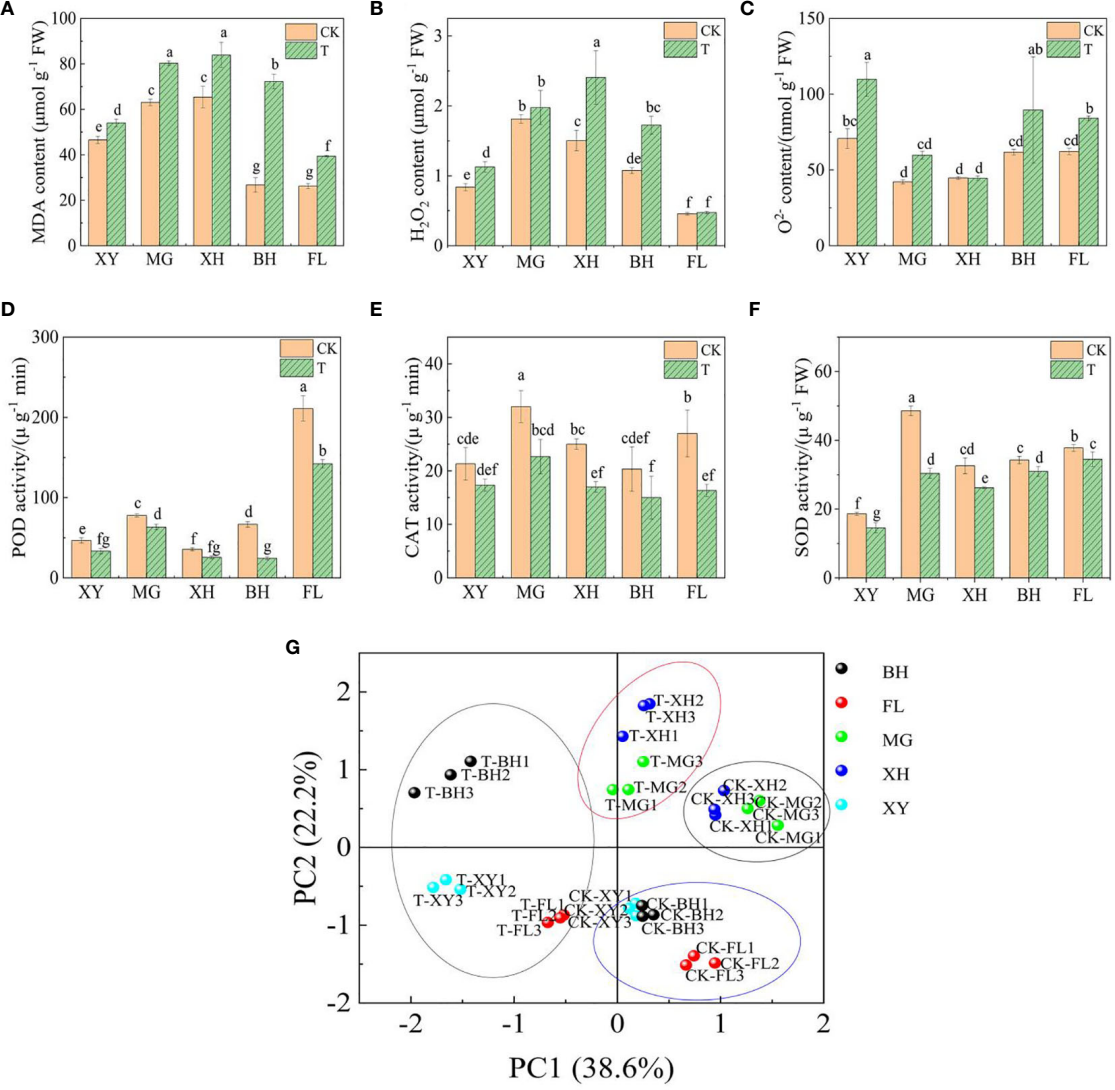
Physiological analysis of five grape varieties under HT stress. The graphs showed the levels of **(A)** malondialdehyde (MDA) content. **(B)** hydrogen peroxide (H_2_O_2_) content. **(C)** Superoxide anion (O^2−^) content. **(D)** peroxidase (POD) activity. **(E)** catalase (CAT) activity. **(F)** Superoxide dismutase (SOD) activity. Values followed by the same letter were not significantly different (p < 0.05) according to Duncan’s assay significant difference test. **(G)** Principal component analysis (PCA) of the five grape leaves. Small letters indicate significance between the different varieties (P <0.05). In subpanels **(A–G)**, CK and T represent the control treatment (35 °C ± 2 °C) and high-temperature treatment (40 °C ± 2 °C), respectively. XY, MG, XH, BH, and FL represent “Xinyu,” “Miguang,” “Summer Black,” “Beihong,” and “Flame seedless” grapes, respectively.

The levels of MDA and H_2_O_2_ increased significantly in XH and BH grapes (**Figures 2A, B**), while O^2−^ content increased in XY and BH grapes (**Figure 2C**), indicating that oxidative damage occurred in the plants. The activities of SOD, POD, and CAT decreased in all five grape varieties after exposure to HT stress (**Figures 2D–F**). This reduction in enzyme activity may be due to structural damage and depletion of antioxidant enzymes. Principal component analysis (PCA) showed that the first and second principal components explained 38.6% and 22.2% of the variance, respectively (**Figure 2G**). Before the high-temperature treatment, the first group of cultivars (BH, XY, and FL grapes) and the second group of cultivars (XH and MG grapes) clustered together. However, after the high-temperature treatment, the five grape varieties showed more significant separation, indicating that HT had different effects on them compared to CK. **Supplementary Figure S1** shows the changes in chlorophyll fluorescence and photosynthetic parameters of leaves from different grape cultivars following the HT stress treatment. Under HT conditions, the total chlorophyll content (Chl), intercellular CO_2_ concentration (Ci), net photosynthetic rate (Pn), transpiration rate (Tr), and stomatal conductance (Gs) decreased. Conversely, the intercellular CO_2_ concentration (Ci) and initial fluorescence (Fo) increased in all five grape cultivars. Notably, the largest increases in Ci and Fo were observed in BH grapes, suggesting that HT stress had the most significant effect on light energy conversion efficiency in this cultivar.

### 3.2 Quality evaluation of RNA-Seq data

The raw data of 30 samples from XY, MG, XH, BH, and FL grapes were analyzed, and it was found that Q20 (%) >97% and Q30 (%) >93% met the standards for further biological analysis. Transcriptome sequencing data from 30 samples were filtered to remove adapters and low-quality reads to obtain Clean Reads and Clean Data. The ratio of clean reads and clean data obtained from each sample to the original reads was above 92%. The filtered reads were aligned to the reference genome using the upgraded version of HISAT2 (http://ccb.jhu.edu/software/hisat2/index.shtml) in TopHat2. At least 36,996,094 reads were aligned with the reference genome for each sample, accounting for more than 93.52% of the total clean reads for each sample. Of these, 97.43%–97.75% of the reads were aligned to a unique position in the reference genome, while 2.25% to 2.57% of the reads were aligned to multiple positions in the reference genome (**Supplementary Table S1**). In general, the RNA-seq data alignment rate was high, indicating that the RNA-Seq data utilization rate was high, which ensured the validity and accuracy of the sequence assembly and post-analysis.

We conducted PCA on the samples and found that the spatial dimensions of the XH and FL samples were similar to those of the control samples after HT treatment; however, the spatial distance was large, indicating that different temperature treatments had a significant impact on the expression of samples (**Supplementary Figure S2**). Secondly, we analyzed the repeatability of RNA-seq samples using Pearson’s Correlation Coefficient (r) as a parameter of biological repeat correlation. The closer the value of r^2^ is to 1, the higher the correlation between the two replicate samples, and the closer it is to 0, the weaker the correlation. In this study, the r^2^ values between the three biological replicates of the same sample were close to 1, indicating that the RNA-Seq samples had good repeatability, and the data were reliable (**Supplementary Figure S3**).

### 3.3 Differential expression in five grape varieties

After analyzing the gene expression of the 10 treatment groups, we compared grape varieties treated at different temperatures with the same grape varieties treated at different temperatures. We used DEGseq to identify genes that were differentially expressed under HT (q-value ≤0.05, |log2 fold-change| ≥1). Hierarchical clustering analysis (HCA) showed that the high-temperature-treated samples and control samples were clustered together, indicating an overlap in responsive genes between these treatments (**Supplementary Figure S4**).

**Figure 3:**
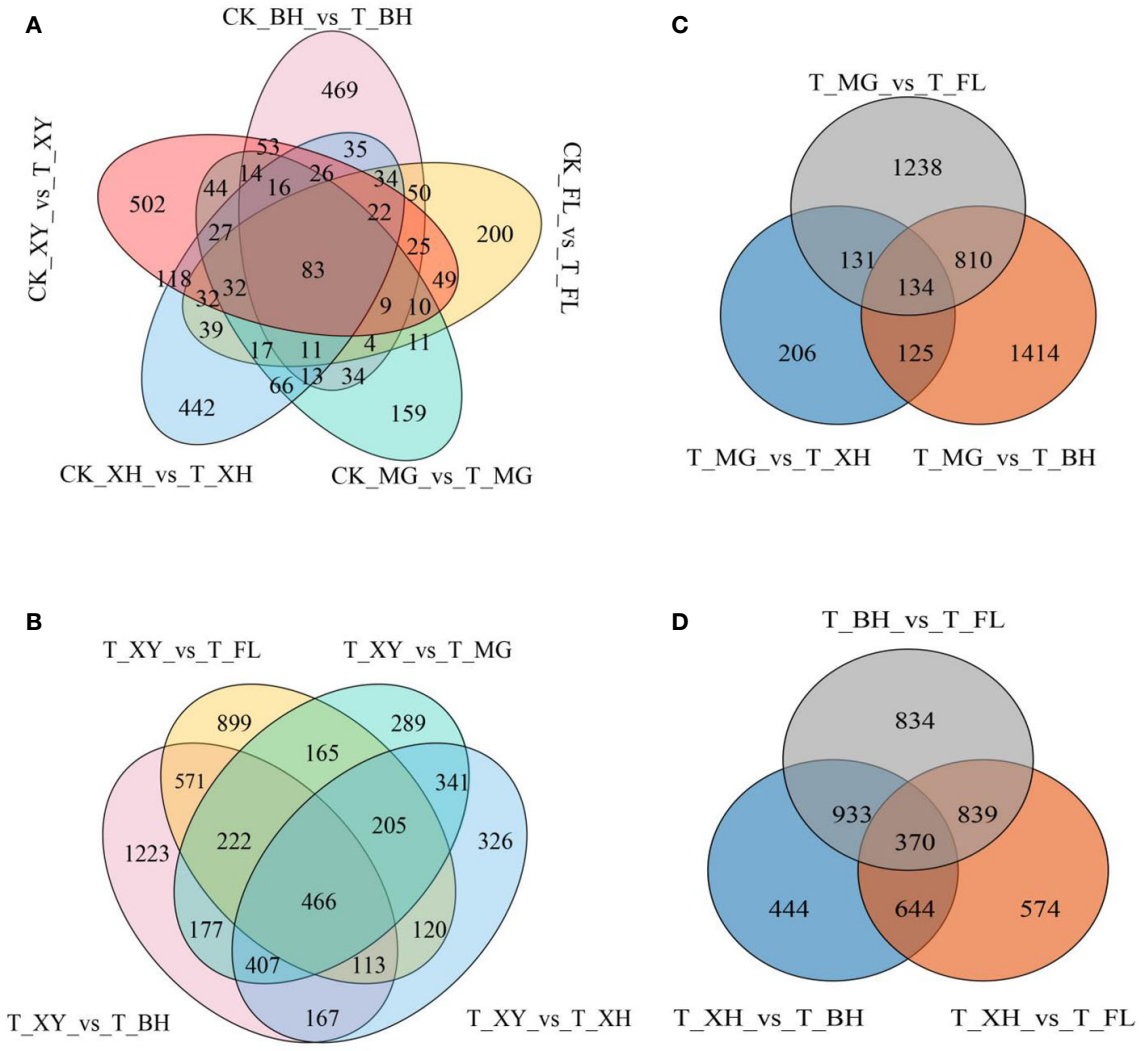
DEG analysis of five grape cultivars after HT treatment. **(A)** Total number of DEGs and the shared core sets of up and downregulated DEGs in all grape cultivars under different temperature treatments. **(B–D)** Statistics of DEGs and shared core sets of up and downregulated DEGs in all grape cultivars under HT treatments. In subpanels **(A–D)**, CK and T represent the control treatment (35 °C ± 2 °C) and high-temperature treatment (40 °C ± 2 °C), respectively. XY, MG, XH, BH, and FL represented “Xinyu,” “Miguang,” “Summer Black,” “Beihong,” and “Flame seedless” grapes, respectively.

In this study, we identified 550 (upregulated 314, downregulated 236), 628 (upregulated 414, downregulated 214), 898 (upregulated 487, downregulated 411), 1,013 (upregulated 557, downregulated 456), and 1,062 (upregulated 461, downregulated 601) genes in XY, MG, XH, BH, and FL grapes, respectively. Furthermore, we observed a significant increase in differentially expressed genes (DEGs) under heat stress in different grape varieties. Among these varieties, MG grapes had the least number of DEGs, while XY grapes had the greatest number (**Supplementary Table S2A**; **Supplementary Figure S5**). We conducted a comparative analysis of DEGs among grape cultivars exposed to different temperatures. Specifically, we identified the intersection of upregulated and downregulated DEGs across all grape cultivars to determine shared core sets. In addition, we examined the intersection of DEGs among grape varieties under HT treatment to identify the shared core sets. In the control group compared to the HT treatment group, we identified 83 DEGs (**Figure 3**). We found that HT stress resulted in a greater number of upregulated than downregulated genes in different grape varieties. A detailed list of the shared DEGs grouped by treatment type is presented in **Supplementary Table S2B**.

### 3.4 Functional enrichment analysis of DEGs in five grape cultivars

**Figure 4:**
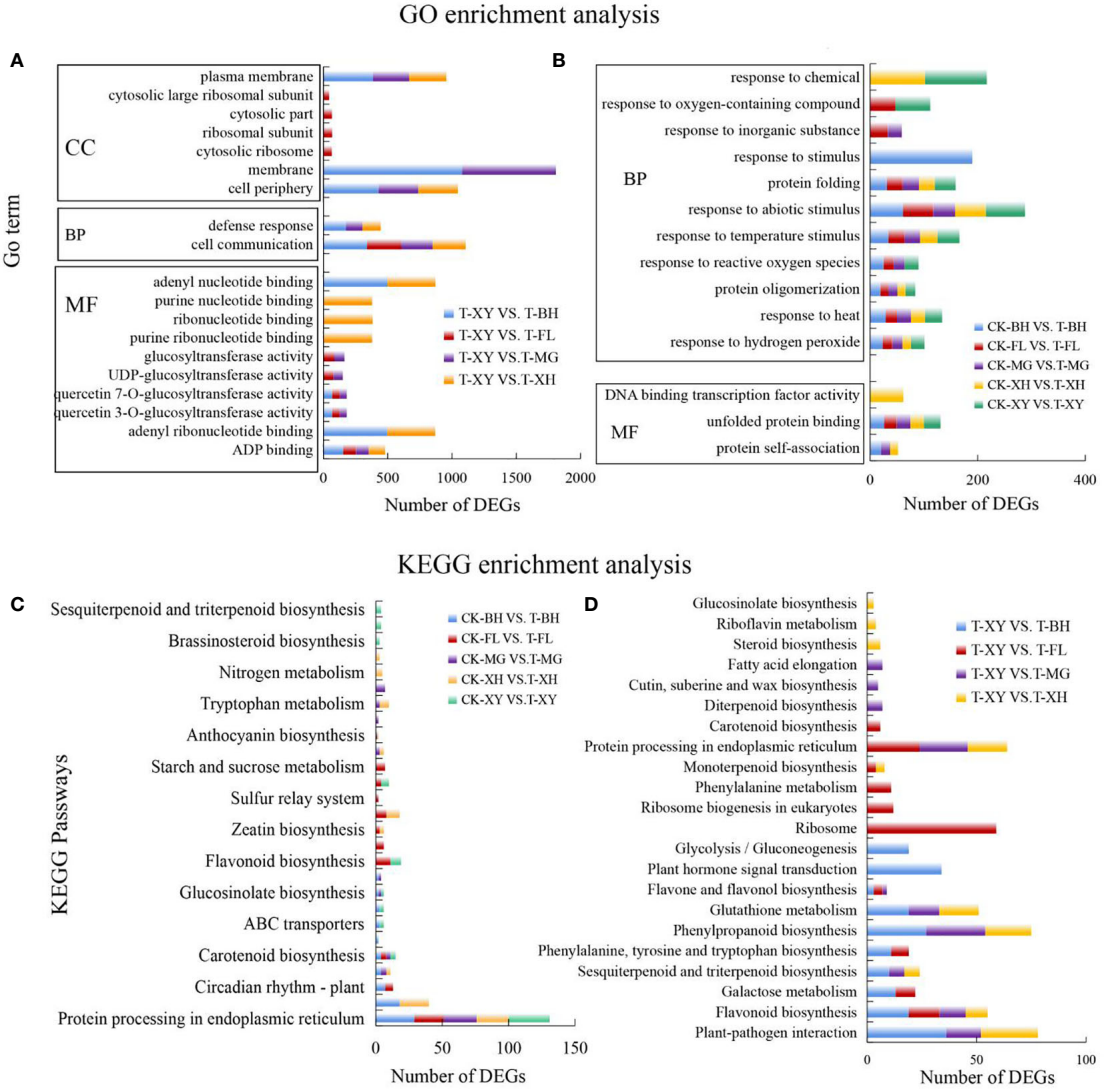
Functional enrichment analysis of differentially expressed genes in the five grape varieties. **(A, B)** GO enrichment analysis was performed for the DEGs. Y is the term of GO, and X is the number of DEGs. MF, molecular function; CC, cellular component; BP, biological process. Select the top 10 GO term entries with the smallest p-value and the most significant enrichment in each Go classification for display. **(C, D)** analyzed the significant enrichment of the differential gene KEGG and selected the top 10 pathways with the smallest p-value and the most significant enrichment for display. In subpanels **(A–D)**, CK and T represent the control treatment (35 °C ± 2 °C) and high-temperature treatment (40 °C ± 2 °C), respectively. XY, MG, XH, BH, and FL represent “Xinyu,” “Miguang,” “Summer Black,” “Beihong,” and “Flame seedless” grapes, respectively.

Through GO enrichment analysis, DEGs were enriched into three categories: biological process (BP), cellular component (CC), and molecular function (MF). In the comparison of the same temperature varieties with different varieties, in the BP category, cell communication and defense responses were significantly enriched. ADP binding, adenyl ribonucleotide binding, and adenyl nucleotide binding were significantly enriched in MF category. In the CC category, the cell periphery, membrane, and plasma membrane were significantly enriched (**Figures 4A, B**). Lists of annotation and enriched GO terms for these DEGs grouped by treatment type can be found (**Supplementary Tables S3A, B**).

To clarify the biochemical metabolism or signal transduction pathways in which different genes may participate in different samples, KEGG pathway enrichment analysis was carried out. In the comparison of five grape varieties under different temperatures, flavonoid biosynthesis, protein processing in the endoplasmic reticulum, plant hormone signal transduction, carotenoid biosynthesis, and starch and sucrose metabolism were significantly enriched (**Figure 4C**). In the comparison of the control with the five grapes, glutathione metabolism, DNA replication, flavonoid biosynthesis, and phenylpropanoid biosynthesis were significantly enriched (**Figure 4D**). Lists of annotation and enriched pathways for these DEGs grouped by treatment type can be found (**Supplementary Tables S4A, B**). These pathways mainly involve were genetic information processing, metabolic pathways, and environmental information processing. HT stress significantly promoted DEGs involved in protein processing in the endoplasmic reticulum pathway, and many HSPs were upregulated. This indicates that grapes can improve their heat tolerance by rapidly accumulating heat shock proteins under HT stress. At the same time, the peroxisome pathway was also activated and upregulated in the five grape varieties, genes involved in photosynthesis and antenna protein were inhibited, and differential genes in the carotenoid biosynthesis pathway were promoted. This may indicate that the growth of the grapes was inhibited. Genes related to glutathione metabolism, proline metabolism, ascorbate and aldate metabolism were upregulated to protect plants.

### 3.5 Expression of antioxidant enzyme-related genes

**Figure 5:**
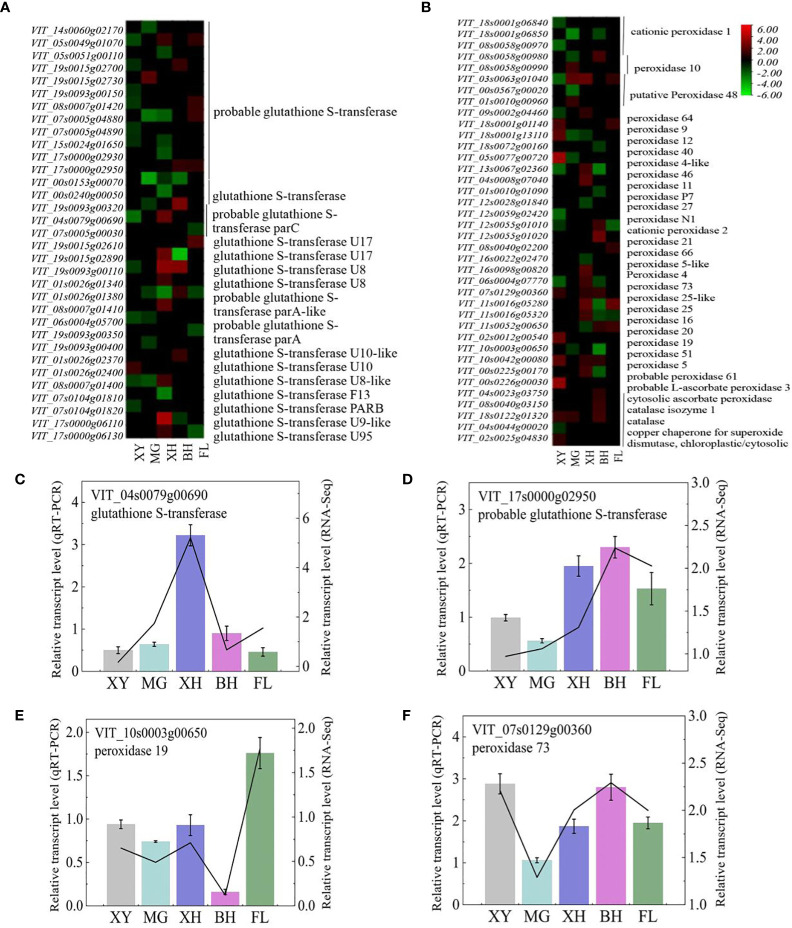
**(A, B)** Heatmap of the expression patterns of the selected genes involved in ROS scavenging in response to HT stress. The color bar indicates gene fold change, upregulation is indicated in red, and downregulation is indicated in green. **(C–F)** Expression profiles of the selected DEGs determined using RT-PCR analyses and the line indicates the relative gene transcription level in RNA-Seq. Error bars represent mean ± SD. XY, MG, XH, BH, and FL represent CK_XY_vs_T_XY, CK_MG_vs_T_MG, CK_XH_vs_T_XH, CK_BH_vs_T_BH, and CK_FL_vs_T_FL, respectively. CK and T represent the control treatment (35 °C ± 2 °C) and high-temperature treatment (40 °C ± 2 °C), respectively.

In this study, 105 genes encoding enzymes were involved in ROS regulation. Two *catalase* (*CATs*), two *ascorbate peroxidase* (*APXs*), 52 *glutathione S-transferase* (*GSTs*), two *superoxide dismutase* (*SODs*), two *glutathione peroxidase* (*GPXs*), and 49 *peroxidase* (*POXs*) genes were differentially expressed after HT treatment compared to the controls (**Figures 5A, B**). These DEGs showed different expression patterns in different grape cultivars under HT stress. After HT treatment, most DEGs were upregulated in BH, FL, and XH grapes, and downregulated in MG and XY grapes. Among the cultivars, there were 15 genes with differential expression of more than 4.00 times. Lists of these DEGs can be found (**Supplementary Table S5**). To validate the RNA-Seq data, four DEGs were selected for real-time PCR analysis (**Figures 5C–F**). The expression patterns of both the qRT-PCR and RNA-Seq data were highly consistent. Therefore, these genes may play an important role in the heat-stress response of grape leaves.

### 3.6 Expression of transcription factor genes

**Figure 6:**
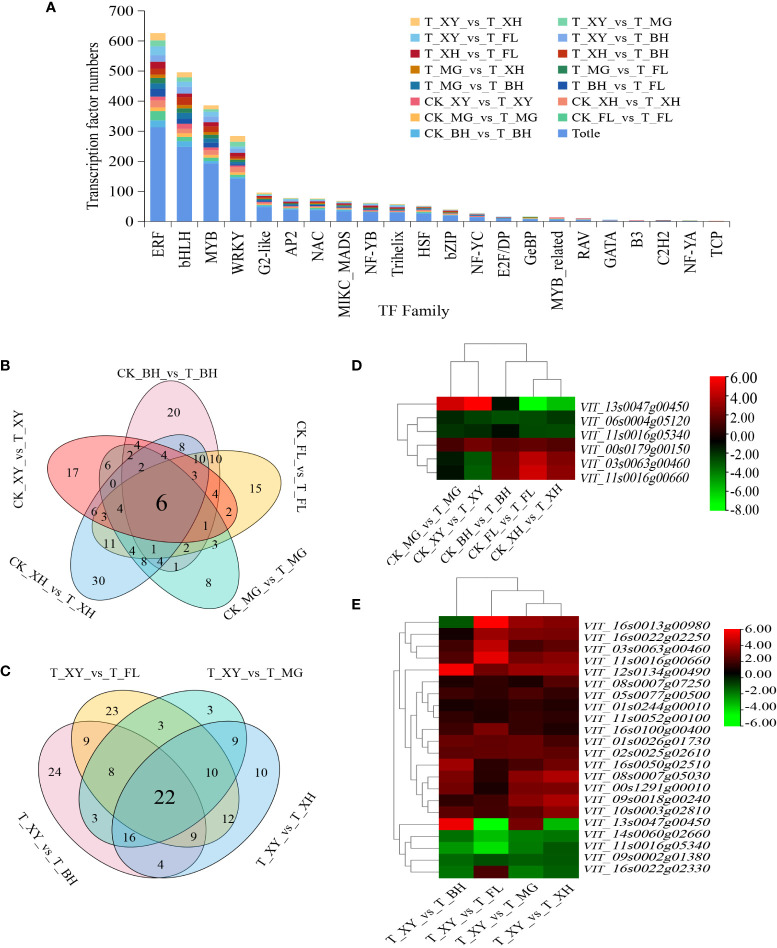
Transcription factor families were differentially expressed in grape cultivars under the HT treatment. **(A)** Statistics of the differential expression of transcription factors in the five grape varieties under HT stress. **(B, C)** Venn clustering analysis was conducted. Shared core sets of up and downregulated TFs in all grape cultivars under HT treatment. **(D, E)** Heat map of core TF expression. Upregulation is indicated in red, and downregulation is indicated in green. In subpanels **(A–E)**, CK and T represent the control treatment (35 °C ± 2 °C) and high-temperature treatment (40 °C ± 2 °C), respectively. XY, MG, XH, BH, and FL represented “Xinyu,” “Miguang,” “Summer Black,” “Beihong,” and “Flame seedless” grapes, respectively.

A total of 1,213 transcripts encoding TFs were differentially expressed in the five grape cultivars. Among the differentially expressed TFs, the bHLH, ERF, MYB, WRKY, G2-like, and HSF families were found to be represented by more than 76% of the TF-encoding transcripts in the five grape cultivars (**Figure 6A**). There were six common transcription factors in CK_BH_vs_T_BH, CK_FL_vs_T_FL, CK_MG_vs_T_MG, CK_XH_vs_T_XH, and CK_XY_vs_T_XY, among which, one *HSF* (*VIT_00s0179g00150*, upregulated in five grape cultivars after HT treatment), three *ERFs* (*VIT_11s0016g05340* upregulated in five grape cultivars after HT treatment, *VIT_03s0063g00460* and *VIT_11s0016g00660* upregulated in BH, FL and XH, but downregulated in MG and XY after HT treatment), one *G2-like* (*VIT_06s0004g05120*, downregulated in five grape cultivars after HT treatment) and one *bHLH* (*VIT_13s0047g00450* downregulated in BH, FL, and XH after HT treatment, but upregulated in MG and XY after HT treatment) (**Figures 6B, D**). In addition, 22 common TFs encoding six *ERFs*, five *bHLHs*, four *MYBs*, four *WRKYs*, one *NF-YB*, one *MIKC_MADS*, and one *AP2* in T_XY_vs_T_BH, T_XY_vs_T_FL, T_XY_vs_T_MG, and T_XY_vs_T_XH. Lists of these DEGs can be found (**Supplementary Table S6**). Among them, 16 DEGs were significantly upregulated after HT treatment and three DEGs were significantly downregulated after HT treatment (**Figures 6C, E**).

In the *HSFs* families, five genes were induced during heat treatment. *HSFA-6b* (*VIT_00s0179g00150*) was significantly upregulated in all five grape cultivars after HT treatment. *HSFA-6b* (*VIT_05s0020g04090*), *HSFAB-2b*, *HSFB-3*, and *HSFBC-1* were significantly downregulated in XY, BH, and FL grapes after HT treatment, but were not significantly expressed in MG grapes. Interestingly, heat significantly upregulated the *bHLHs* and *WRKYs* genes after HT treatment. Several TFs, including *ERFs* and *MYBs*, were specifically downregulated after HT treatment. The basic leucine zipper (*bZIPs*), *NACs*, *C2H2s* genes were heat-regulated in grape leaves and showed different expression patterns in five grape cultivars after HT treatment.

### 3.7 Metabolic pathways under HT stress

#### 3.7.1 Abscisic acid

**Figure 7:**
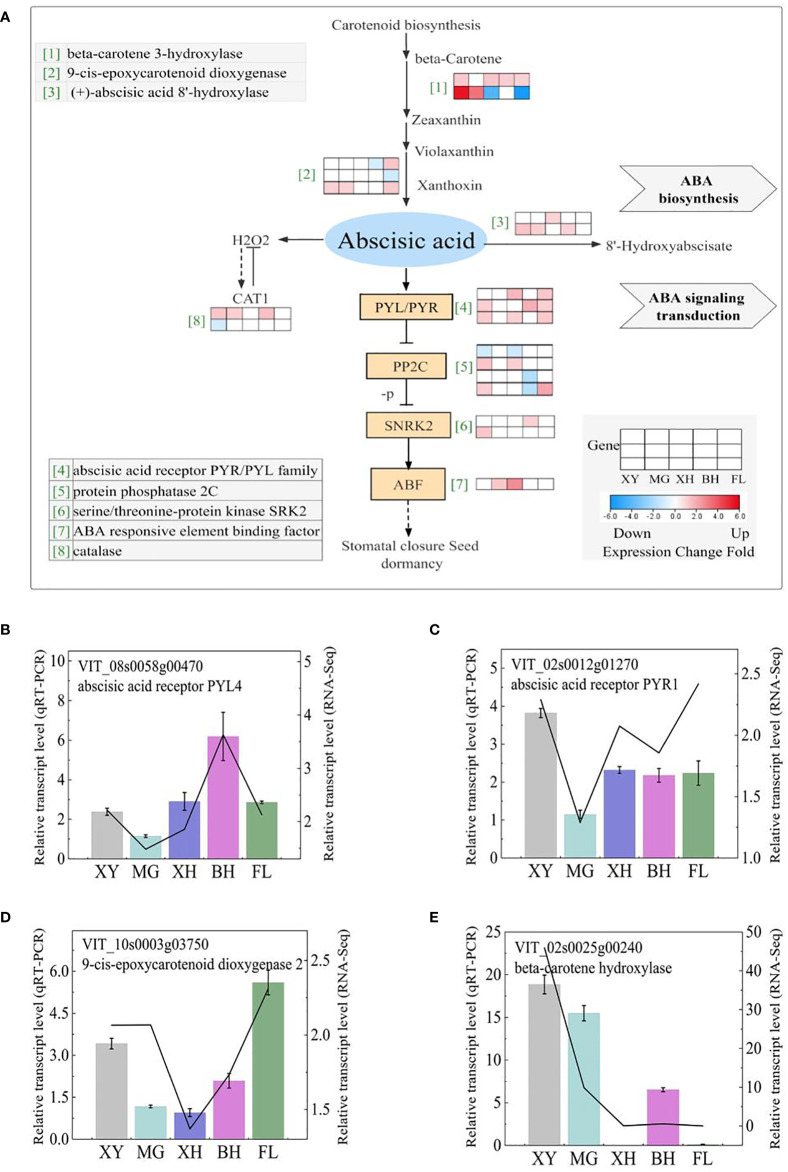
ABA biosynthesis and signaling transduction demonstrating a logfold change in DEGs because of the HT treatment. **(A)** ABA biosynthesis and signaling transduction pathways. Each row represents a significant DEG. The maximum and minimum values of gene expression in the same row are given a corresponding color. Genes without colors were not differentially expressed in this experiment. **(B–E)** Expression profiles of the selected DEGs determined using RT-PCR analyses and the line indicates the relative gene transcription level in RNA-Seq. Error bars represented the mean ± SD. XY, MG, XH, BH, and FL represent CK_XY_vs_T_XY, CK_MG_vs_T_MG, CK_XH_vs_T_XH, CK_BH_vs_T_BH, and CK_FL_vs_T_FL, respectively. CK and T represent the control treatment (35 °C ± 2 °C) and high-temperature treatment (40 °C ± 2 °C), respectively.

In this study, two pathways representing components of ABA, carotenoid biosynthesis, and ABA signal transduction (**Figure 7A**), were found to be significantly enriched after HT treatment. Four DEGs correlated with ABA biosynthesis and signal transduction were selected for qRT-PCR analysis (**Figures 7B–E**), and the expression patterns of qRT-PCR and RNA-Seq were highly consistent. Additional information on these genes can be found in **Supplementary Table S7**. ABA biosynthesis was significantly enriched with two *beta-carotene 3-hydroxylase* (*crtZ*) genes and three *9-cis-epoxycarotenoid dioxygenase* (*NCEDs*) genes. Furthermore, *beta-carotene hydroxylase* was found to be significantly upregulated in XY and MG but downregulated in XH and FL.

The expression of genes associated with the ABA catabolic process, including three *(+)-abscisic acid 8’-hydroxylase* (*CYP707A*). Notably, the interaction between ABA and H_2_O_2_, ABA promoted H_2_O_2_, and *CAT* gene expression can inhibit H_2_O_2_. Except for MG, three ABA receptor-encoding genes *PYL* and four *protein phosphatase 2c* (*PP2Cs*) were significantly expressed in the other four grape cultivars, as were two *serine/threonine-protein kinase* (*SRK2s*) genes and one ABA-responsive element binding factor (*ABF*) gene associated with ABA signaling transduction.

#### 3.7.2 Auxin

**Figure 8:**
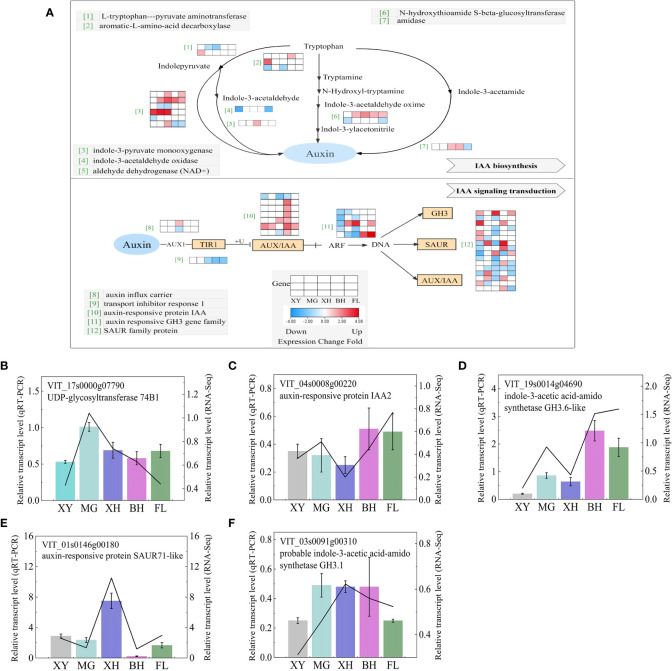
IAA biosynthesis and signaling transduction demonstrating a logfold change in DEGs because of HT treatment. **(A)** IAA biosynthesis and signaling transduction pathways. Each row represented a significantly DEG. The maximum to minimum values of gene expression in the same row were given a corresponding color. Genes without color were not differentially expressed in this experiment. **(B–F)** Expression profiles of the selected DEGs determined using RT-PCR analyses and the line indicates the relative gene transcription level in RNA-Seq. Error bars represented the mean ± SD. XY, MG, XH, BH, and FL represent CK_XY_vs_T_XY, CK_MG_vs_T_MG, CK_XH_vs_T_XH, CK_BH_vs_T_BH, and CK_FL_vs_T_FL, respectively. CK and T represent the control treatment (35 °C ± 2 °C) and high-temperature treatment (40 °C ± 2 °C), respectively.

The transcription of the auxin pathway, including auxin synthesis and signal transduction, was very different among the five grape varieties after heat stress (**Figure 8A**); the information on these genes is shown in **Supplementary Table S8**. Among the four pathways of ethylene production, the tryptophan–indolepyruvate–IAA pathway enriched the most DEGs, including two *L-tryptophan-pyruvate aminotransferase* (*TAA1*) genes and six *indole-3-pyruvate monooxygenase* (*YUCCAs*) genes. XH grapes enriched the most DEGs. Of these, four genes were upregulated and two genes were downregulated. BH grape had the least enriched DEGs, with only two genes. It has been speculated that heat stress mainly affects auxin synthesis in grapes through this pathway. Genes associated with IAA signal transduction were the most affected by HT and tended to show complex expression patterns among the five grape varieties. Seven *auxin-responsive protein IAA* genes, 33 *small auxin up RNA (SAUR)* family protein genes and five *gretchen hagen 3* (*GH3*) family genes were significantly enriched. Among them, XH enriched the most downregulated genes. BH enriched more upregulated genes and MG enriched the least DEGs. To validate the RNA-Seq data, five DEGs that correlated IAA biosynthesis and signal transduction were selected for qRT-PCR analysis (**Figures 8B–F**), and the expression patterns of both qRT-PCR and RNA-Seq were highly consistent.

#### 3.7.3 Starch and sucrose metabolism

**Figure 9:**
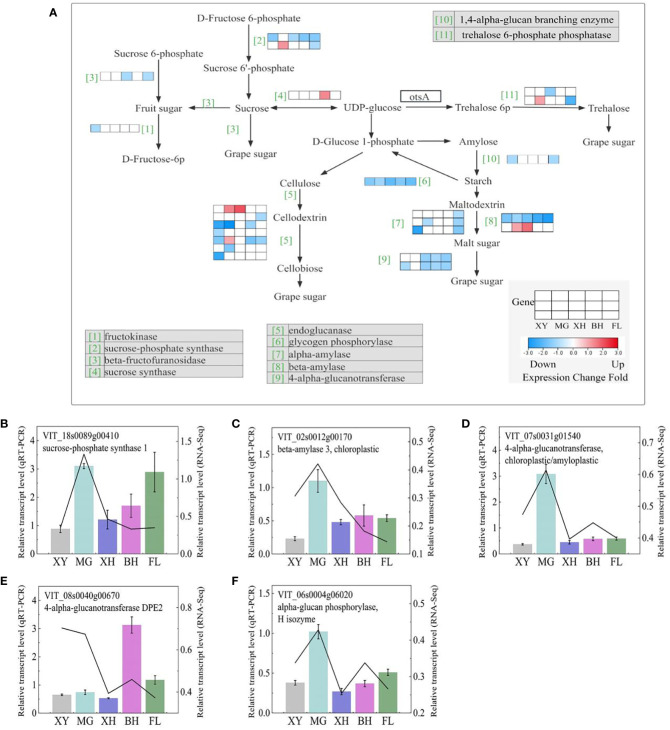
Starch and sucrose metabolism demonstrating major shifts in the expression of metabolic enzymes in response to HT stress in the five grape cultivars. **(A)** Starch and sucrose metabolism pathways. Each row represents a significant DEGs. The maximum to minimum values of gene expression in the same row were given a corresponding color. Genes without color were not differentially expressed in this experiment. **(B–F)** Expression profiles of the selected DEGs determined using RT-PCR analyses the line indicated the gene relative transcription level in RNA-Seq. Error bars represent mean ± SD. XY, MG, XH, BH, and FL represent CK_XY_vs_T_XY, CK_MG_vs_T_MG, CK_XH_vs_T_XH, CK_BH_vs_T_BH, and CK_FL_vs_T_FL, respectively. CK and T represent the control treatment (35 °C ± 2 °C) and high-temperature treatment (40 °C ± 2 °C), respectively.

We observed that all HT treatments resulted in the major downregulation of genes leading to the production of d-fructose, d-glucose 6-phosphate, d-glucose, and alpha-trehalose (**Figure 9A**). Three *sucrose synthase* (*SUSs*) genes were upregulated in T_XY_vs_T_FL, and two *sucrose-phosphate synthase* (*SPSs*) genes were downregulated in four grapes, but not in MG grapes, and the information on these genes is shown in **Supplementary Table S9**. Four *alpha-amylase* (*AMYs*), three *beta-amylase*, one *glycogen phosphorylase* (*PYG*), and two *4-alpha-glucanotransferase* (*malQs*) genes were downregulated in five grapes after HT treatment (**Supplementary Table S9**). HT treatment resulted in different expression patterns of genes involved in the conversion of UDP-glucose to d-fructose, d-glucose, and maltose.

Simultaneously, five DEGs that correlated starch and sucrose metabolism were selected for qRT-PCR analysis (**Figures 9B–F**), and the expression patterns of both qRT-PCR and RNA-Seq were highly consistent.

### 3.8 Physiological index-related DEGs revealed by analysis of co-expression networks

**Figure 10:**
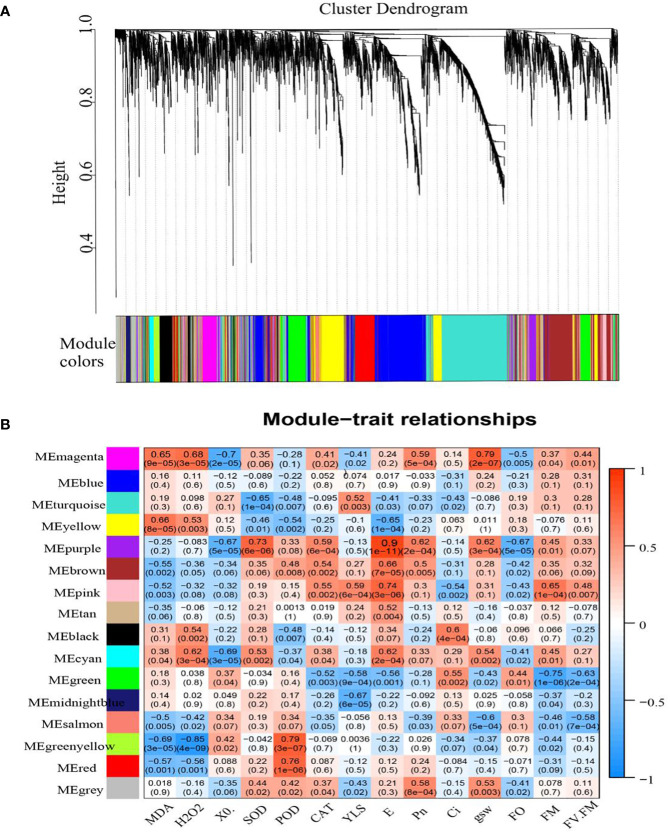
WGCNA of DEGs in grape leaves subjected to HT. **(A)** Hierarchical clustering of samples. Each color in the figure indicates that each gene in the clustering tree corresponding to one color belongs to the same module. **(B)** Module—biological character correlations and corresponding p-values. The left panel shows 16 modules. The color scale on the right displays a module-trait correlation from −1 (blue) to 1 (red).

A total of 16 WGCNA modules were identified using co-expression network analysis (**Figures 10A, B**). Among them, the MEpurple module, composed of 167 genes, had the highest correlation with the transpiration rate. The other modules showed lower correlations. There were 17 genes in the MEpurple module (seven *HSP20s*, three *HSPA1s*, two *HSPE1s*, two *HSP1s*, two *HSP90Bs*, and one *HSPA9*) and one *heat shock transcription factor* (*HsfA2*). These DEGs were significantly upregulated after high-temperature treatment compared with the controls (**Supplementary Figure S6**).

GO enrichment analysis of MEpurple module genes mainly includes “response to temperature stimulus,” “response to heat,” “unfolded protein binding,” “response to abiotic stimulus,” and “response to reactive oxygen species” terms (**Supplementary Figure S7A**). By comparing the enrichment analysis with KEGG data, the MEpurple module covered pathways related to, “Spliceosome,” “Protein processing in endoplasmic reticulum,” and “Endocytosis” (Q <0.05) (**Supplementary Figure S7B**).

In addition, we established a MEpurple gene regulation network using WGCNA. The results showed that *VIT_06s0004g00240*, *VIT_05s0051g00340*, *VIT_01s0011g04990*, and *VIT_12s0057g00670* were mainly involved in folding, sorting, and degradation (**Supplementary Figure S8**).

## 4 Discussion

HT stress has been shown to significantly reduce grape yield, leading to a lower economic income. We investigated the physicochemical and transcriptomic changes that occur under these conditions to understand the impact of HT on different heat-sensitive grape varieties.

### 4.1 Phenotypic response to HT treatments

Under normal conditions, the ROS content in plants is low, which plays an important role in maintaining the stability of the intracellular signaling system (Schneider et al., 2019; Nadarajah, 2020). After HT stress, plants produced a large amount of ROS. This aggravates the degree of membrane lipid peroxidation and leads to cell death, thus inhibiting plant growth (Qi et al., 2010). Important antioxidant enzymes in plant cells include SOD, CAT, and POD. Antioxidant enzymes can remove H_2_O_2_ and O^2−^ produced by HT stress to maintain ROS in cells and protect the stability of cell membranes. SOD can convert O^2−^ into H_2_O_2_ and O_2_, whereas POD and CAT can further decompose H_2_O_2_ into H_2_O and O_2_ (Ruelland and Zachowski, 2010). In this study, the H_2_O_2_, O^2−^, and MDA contents in the five grape leaves increased significantly after HT treatment. BH and XH accumulated higher levels of H_2_O_2_ and MDA, which may lead to damage to the cell membrane. However, XY and FL were significantly lower than those of the other cultivars. An increase in MDA and H_2_O_2_ content caused by HT stress has also been reported in other plants, such as rice and pepper (Kumar et al., 2012; Li et al., 2015). In this study, the enzyme activities of SOD, CAT, and POD decreased in the five cultivars. This indicated that HT stress inhibited the expression of the enzymes or destroyed their structure of the enzymes, causing a decrease in the activities of the protective enzymes.

### 4.2 Gene transcription profile in response to HT stress

We performed mRNA sequencing to reveal differences in gene expression among the five grape varieties. The five grape cultivars exhibited distinct differences in their transcriptome levels in response to heat stress. After HT stress, T_XY_VS_T_BH and CK_XY_VS_T_XY showed the highest numbers of DEGs. A previous study also found that the number of DEGs was far greater in heat-resistant jujube and *Pyropia haitanensis* strains than in the heat-sensitive jujube and *P. haitanensis* strains (Wang et al., 2018; Jin et al., 2020). In our study, XY was better able to increase transcriptional regulation in response to HT stress than BH grapes. Through GO and KEGG enrichment analysis, these DEGs were mainly enriched in “response to temperature stimulus,” “response to active oxygen specifications,” “response to heat,” “response to hydrogen peroxide,” “response to abiotic stimulus,” “protein processing in endogenous reticulum,” “plant hormone signal transformation,” and “cartotenoid biosynthesis.” This may reflect their similar responses to heat stress, which is similar to previous studies (Jin et al., 2020; Liu M. et al., 2020). This indicated that similar key regulatory pathways play important roles in the response to high-temperature stress.

### 4.3 HSPs and transcription factors related to HT stress response

HSPs are important cellular response proteins in plants that can be immediately induced by HT (Liu et al., 2012; Wang et al., 2019). HSP70 and HSP90 have been identified as heat response factors in tomatoes, dates, and rice (Frank et al., 2009; Jung et al., 2012; Jin et al., 2020). The expression levels of *HSPs* (*HSP17.6*, *HSP22*, *HSP21*, and *HSP40*) are significantly increased in grapevine leaves under heat stress (Wang et al., 2010; Jiang et al., 2017). In this study, a total of 36 *HSPs* chaperones were significantly up regulated by HT stress, including *HSP101*, *HSP70*, *HSP83*, *HSP90*, *HSP18.1*, *HSP17.3*, *HSP17.1*, and *HSP25.3.* These results indicate that upregulated HSP genes play an important role in the heat tolerance of grapes.

TFs are important for regulating plant development and stress responses (Amorim et al., 2017; Ng et al., 2018). Under HT stress, heat stress-induced or heat-suppressed TFs in plants potentially contribute to the differential regulation of downstream genes (Wahid et al., 2007). HSF is closely related to the regulation of plant thermal stress and has been reported in *Arabidopsis*, rice, tomato, and potato (Song et al., 2016; Ibanez et al., 2017; Singh et al., 2018; Fragkostefanakis et al., 2019). *HSFA2* regulates the expression of some *HSPs* genes, such as *HSP101*, *HSP70*, and *HSP15.7* (Nishizawa et al., 2006; Jiang et al., 2017). We found 15 *HSFs* genes, but only four genes were induced during heat treatment. *HSFA2* and *HSFA-6b* were significantly upregulated in five grapes, similar to previous studies (Liu et al., 2012; Jiang et al., 2017). At present, research on *WRKY* transcription factor under HT stress has mainly focused on *Arabidopsis* (Li et al., 2011), wheat (He et al., 2016), and pepper (Cai et al., 2015). We found that *WRKY33*, which could be involved in the heat response of grape leaves, was upregulated in three grape cultivars. Overexpression of *MYB* genes can enhance the thermotolerance of genetic plants (Amano et al., 2012; Zhao et al., 2017). Wang et al. (2019) found that the expression of some *MYB* transcription factors changed after heat stimulation in grape leaves. Our results indicated that *MYBA6* and *MYB108-like* are induced by HT. However, *MYB114* was strongly repressed in five grapes, indicating that they were involved in the HT stress response. The overexpression of *TaNAC2L* and *NAC019* can improve thermotolerance in *Arabidopsis* (Guan et al., 2014; Guo et al., 2015). *NAC56* was highly expressed in MG grapes, but downregulated in other grapes, suggesting that *NAC56* participates in the heat stress response and plays different roles in heat-resistant and heat-sensitive cultivars. *bHLH* plays an important role in plant stress resistance. However, there are few reports on the response of *bHLH* cells to HT stress. In our study, many *bHLH* genes were significantly upregulated after heat treatment, which is consistent with reports on tea (Wang et al., 2019) and wheat (Cui et al., 2018).

### 4.4 Endogenous hormone pathways in response to HT

Abiotic and biotic stresses are effectively responded to by abscisic acid (ABA) and is called “stress hormone” (Suzuki et al., 2016; Zhu, 2016). Adversity stress leads to a rapid increase in ABA content in plants, which enhances ABA signaling and thus improves stress resistance (Ji et al., 2011). A key rate-limiting enzyme in ABA biosynthesis is 9-cis-epoxycarotenoid dioxygenase (NCED) (Hwang et al., 2018), which has an obvious regulatory effect on abiotic stresses, such as drought (Li et al., 2018) and heat stress (Zhou et al., 2022). In our study, *NCED2* was greatly upregulated in three grapes and *NCED1* was upregulated in one grape after HT treatment. Drought stress has been reported to be triggered by PYR/PYL/RCAR (hereafter PYLs) proteins. These proteins function as ABA receptors (Pizzio et al., 2013; Liu et al., 2019). Two *PYL4s* and one *PYR1* were upregulated after HT in the four grape cultivars. This indicates that, under HT stress, the *VvPYL4* gene is involved in the defense response induced by HT. Protein phosphate 2C (PP2C), a key regulator of the ABA signaling pathway, also has an obvious regulatory effect on abiotic stress (Manabe et al., 2007; Bhaskara et al., 2017; Lenka et al., 2018). In this study, one *PP2C* and four probable *PP2Cs* were upregulated and downregulated, respectively, after HT in the four grape cultivars, and there was no differential expression in MG grapes.

In this study, we found that the expression patterns of genes related to IAA synthesis were significantly altered in plants exposed to heat stress. After HT stress, most *GH3* genes were downregulated in CK_MG_vs_T_MG, CK_XH_vs_T_XH, and CK_XY_vs_T_XY. This is consistent with the results of previous studies (Du et al., 2013). Aux/IAA proteins mediate drought tolerance in Arabidopsis by regulating glucosinolate levels (Salehin et al., 2019). *IAA5*, *IAA6*, and *IAA19* are important for drought responses (Shani et al., 2016). Plants overexpressing OsIAA20 showed the opposite phenotype to that of OsIAA20 RNAi transgenic rice (Zhang et al., 2021). After HT treatment, *IAA9* was upregulated in the four grape cultivars, and the upregulation in the expression of *IAA28* was BH grape-specific.

### 4.5 Sugar and starch

Many crop species have been shown to be stressed by abiotic stresses related to sugars (Parrotta et al., 2016; Londo et al., 2018; Wang et al., 2020). In higher plants, sucrose synthase (SuS) and sucrose phosphate synthase (SPS) are the key enzymes involved in sucrose metabolism. A recent study found that *SUS* and *SPS* might participate in resistance to HT stress (Zhou et al., 2016; Kana et al., 2018; Verma et al., 2018). The HT treatment repressed the expression of *SUS7* and *SPS1* in the five grape cultivars. When stress was present, SUS and Invertase (INV) levels decreased before ABA levels increased, and stress-induced increases in ABA further inhibited their expression. (Ruan, 2014). HT treatment resulted in the following results: *SPS*, *SUS*, and *INV*-related genes were significantly downregulated in CK_FL_vs_T_FL and CK_XY_vs_T_XY, indicating that HT inhibited sucrose metabolism in XY and FL grapes, whereas XY grapes had a significantly increased sensitivity to HT in their sucrose metabolism.

Alpha-amylase (AMY) and beta-amylase are the main enzymes involved in the catabolism of starch. In our study, the expression of four AMY and two beta-amylase genes encoding enzymes that degrade starch into maltose and dextrin in five grape varieties was downregulated after HT treatment. Two *4-alpha-glucanotransferases* (*malQ*) responsible for the degradation of malt sugar into grape sugar were also downregulated, suggesting that HT stress inhibited starch degradation, thereby repressing the accumulation of grape sugar, especially in FL grapes. The enzyme cellulase hydrolyzes cellulose to glucose, which is then degraded by endoglucanase (EGL) (Payne et al., 2015; Chylenski et al., 2019). AnEGL demonstrated salt tolerance and thermostability in high salinity environments (Cai et al., 2022). Interestingly, six *EGLs* responsible for the degradation of cellulose into cellodextrin and cellobiose were also downregulated in the five grapes after HT treatment, suggesting that cellobiose may be the terminal response to heat. Compared with other grape cultivars, these *EGLs* genes were significantly upregulated, indicating that cellulose degradation in XY grapes is more sensitive to HT. Overall, all heat stresses in the five grape cultivars decreased the production of simple soluble sugars used in general metabolism, likely resulting in reduced growth and development.

### 4.6 Protein processing in endogenous reticulum

When plants are exposed to abiotic stresses, the endoplasmic reticulum plays a major role (Liu et al., 2021; Reyes-Impellizzeri and Moreno, 2021; Cao et al., 2022). Arabidopsis antioxidant defenses are modulated by ROS signaling induced by ER stress (Ozgur et al., 2014). Tomato expressing ER-sHSP constitutively displayed improved salinity tolerance (Fu et al., 2016). In our research, 41 *HSPs* participated in the protein processing in endoplasmic reticulum and significantly up-regulated expression in five grape cultivars after HT treatment, indicating that *ER-sHSP* actively responds to HT treatment to alleviate the damage of HT to grapes. Lists of these DEGs can be found (**Supplementary Table S11**). Calreticulin (CRT) and protein disulfide isomerase (PDI) are molecular chaperones that is involved in the pivotal protein folding in ER, they had been reported about abiotic stress (Jia et al., 2008; Xiang et al., 2015; Wang et al., 2017; Feldeverd et al., 2020; Wai et al., 2020; Meng et al., 2021). After HT stress, two *CRTs* were differentially expressed, of which *VIT_14s0060g01290* was upregulated in CK_FL_vs_T_FL, while *VIT_07s0005g01390* was differentially expressed among grape cultivars (**Supplementary Table S11**). One *PDI* gene (*VIT_12s0059g01560*) was found to increase expression levels in CK_FL_vs_T_FL and CK_XY_vs_T_XY under HT stress (**Supplementary Table S11**), this was consistent with previous research results (Wang et al., 2017; Feldeverd et al., 2020). In summary, *sHSP*, *CRT*, and *PDI* participated in the processing of proteins in the endoplasmic reticulum, affecting grape antioxidant defense and leading to changes in heat resistance.

## 5 Conclusions

In this study, BH and XH grapes accumulated higher levels of H_2_O_2_ and MDA after HT treatment, causing oxidative damage to plants. At the same time, the maximum increases in Ci and Fo were observed in BH grapes. However, XY and FL grapes were significantly lower than the other varieties, indicating that XY and FL grapes were better adapted to this HT environment than BH and XH grapes, followed by MG grapes. Meanwhile, with the help of RNA-Seq analysis, we investigated the underlying mechanisms associated with grape cultivar heat stress responses. We identified 83 shared genes between natural environment control conditions and high-temperature stress treatments across all five grape cultivars. We found that HT treatment resulted in a greater number of upregulated than downregulated genes in different grape varieties. GO and KEGG analyses revealed that DEGs in response to heat stress were enriched in metabolic pathways, protein processing in the endoplasmic reticulum, plant hormone signal transduction, and starch and sucrose metabolism. HT treatment significantly promoted DEGs involved in protein processing in the endoplasmic reticulum pathway, and many HSPs were upregulated. This indicated that grape can improve its heat tolerance by rapidly accumulating heat shock proteins under HT treatment. Our study indicates that examining the HT treatment response of grapevines at 40 °C does not fully elucidate or identify the most important elements of the heat stress response. At 35 °C and 40 °C, we observed very different transcriptional landscapes for key hormones, transcription factors, and sugar pathways under natural temperature and HT conditions. Based on these results, we may gain a better understanding of the molecular mechanisms of the grape heat stress response.

## Data availability statement

The data presented in the study are deposited in the NCBI SRA database, accession link: https://www.ncbi.nlm.nih.gov/sra/PRJNA1053489.

## Author contributions

FD: Data curation, Writing – original draft, Writing – review & editing. FP: Methodology, Writing – review & editing. GL: Investigation, Supervision, Writing – review & editing. JZ: Investigation, Supervision, Writing – review & editing. LZ: Data curation, Writing – review & editing. YW: Data curation, Supervision, Validation, Writing – review & editing. HL: Conceptualization, Funding acquisition, Project administration, Resources, Supervision, Writing – review & editing.

## References

[B1] Amano M. Iida S. Kosuge K. (2012). Comparative studies of thermotolerance: different modes of heat acclimation between tolerant and intolerant aquatic plants of the genus Potamogeton. Ann. Bot. 109 (2), 443. doi: 10.1093/aob/mcr300 22147547 PMC3268545

[B2] Amorim L. L. B. dos Santos R. D. Neto J. P. B. Guida-Santos M. Crovella S. Benko-Iseppon A. M. (2017). Transcription factors involved in plant resistance to pathogens. Curr. Protein Pept. Sci. 18, 335–351. doi: 10.2174/1389203717666160619185308 27323805

[B3] Bhaskara G. B. Wen T. Nguyen T. T. Verslues P. E. (2017). Protein phosphatase 2Cs and *Microtubule-Associated stress protein 1* control microtubule stability, plant growth, and drought response. Plant Cell 29 (1), 169–191. doi: 10.1105/tpc.16.00847 28011693 PMC5304354

[B4] Bineau E. Diouf I. Carretero Y. Duboscq R. Bitton F. Djari A.. (2021). Genetic diversity of tomato response to heat stress at the QTL and transcriptome levels. Plant J. 107 (4), 1213–1227. doi: 10.1111/tpj.15379 34160103

[B5] Bita C. E. Gerats (2013). Plant tolerance to HT in a changing environment: scientific fundamentals and production of heat stress-tolerant crops. Front. Plant Sci. 4. doi: 10.3389/fpls.2013.00273 PMC3728475 23914193

[B6] Blair E. J. Bonnot T. Hummel M. Hay E. Marzolino J. M. Quijada I. A.. (2019). Contribution of time of day and the circadian clock to the heat stress responsive transcriptome in *Arabidopsis* . Sci. Rep. 9 (1), 1–12. doi: 10.1038/s41598-019-41234-w 30886204 PMC6423321

[B7] Cai H. Y. Yang S. Yan Y. Xiao Z. L. Cheng J. B. Wu J.. (2015). CaWRKY6 transcriptionally activates CaWRKY40, regulates Ralstonia solanacearum resistance, and confers high-temperature and high-humidity tolerance in pepper. J. Exp. Bot. 66, 3163–3174. doi: 10.1093/jxb/erv125 25873659

[B8] Cai L. N. Xu S. N. Lu T. Lin D. Q. Yao S. J. (2022). Salt-tolerant and thermostable mechanisms of an endoglucanase from marine aspergillus niger. Bioresour. Bioprocess. 9, 44. doi: 10.1186/s40643-022-00533-3 PMC10991132 38647856

[B9] Cao J. Wang C. Hao N. Fujiwara T. Wu T. (2022). Endoplasmic reticulum stress and reactive oxygen species in plants. Antioxidants (Basel). 11 (7), 1240. doi: 10.3390/antiox11071240 35883731 PMC9311536

[B10] Charng Y. Y. Liu H. C. Liu N. Y. Hsu F. C. Ko S. S. (2006). Arabidopsis Hsa32, a novel heat shock protein, is essential for acquired thermotolerance during long recovery after acclimation. Plant Physiol. 140 (4), 1297–1305. doi: 10.1104/pp.105.074898 16500991 PMC1435801

[B11] Cheng Y. Cheng L. Mi Y. H. Duan H. P. Cha Y. S. Shao J. L.. (2018). Comparative study on various methods for determination of activity of antioxidant enzymes in rice. Jiangxi Agric. J. 30 (02), 108–111. doi: 10.19386/j.cnki.jxnyxb.2018.02.23

[B12] Chylenski P. Bissaro B. Sørlie M. Røhr Å. K. Várnai A. Horn S. J.. (2019). Lytic polysaccharide monooxygenases in enzymatic processing of lignocellulosic biomass. ACS Catal. 9 (6), 4970–4991. doi: 10.1021/acscatal.9b00246

[B13] Cui X. Wang Y. X. Liu Z. W. Wang W. L. Li H. Zhuang J. (2018). Transcriptome-wide identification and expression profile analysis of the bHLH family genes in *Camellia sinensis* . Funct. Integr. Genomics 18 (5), 489–503. doi: 10.1007/s10142-018-0608-x 29651641

[B14] Du H. Liu H. B. Xiong L. Z. (2013). Endogenous auxin and jasmonic acid levels are differentially modulated by abiotic stresses in rice. Front. Plant Sci. 4. doi: 10.3389/fpls.2013.00397 PMC3793129 24130566

[B15] Feldeverd E. Porter B. W. Yuen C. Y. L. Iwai K. Carrillo R. Smith T.. (2020). The arabidopsis protein disulfide isomerase subfamily m isoform, PDI9, localizes to the endoplasmic reticulum and influences pollen viability and proper formation of the pollen exine during heat stress. Front. Plant Sci. 11. doi: 10.3389/fpls.2020.610052 PMC7802077 33447253

[B16] Fraga H. García de Cortázar Atauri I. Malheiro A. C. Santos J. A. (2016). Modelling climate change impacts on viticultural yield, phenology and stress conditions in Europe. Global Change Biol. 22 (11), 3774–3788. doi: 10.1111/gcb.13382 27254813

[B17] Fragkostefanakis S. Simm S. El-Shershaby A. Hu Y. J. Bublak D. Mesihovic A.. (2019). The repressor and co-activator *HsfB1* regulates the major heat stress transcription factors in tomato. Plant Cell Environ. 42, 874–890. doi: 10.1111/pce.13434 30187931

[B18] Frank G. Pressman E. Ophir R. Althan L. Shaked R. Freedman M.. (2009). Transcriptional profiling of maturing tomato (*Solanum lycopersicum* L.) microspores reveals the involvement of heat shock proteins, ROS scavengers, hormones,and sugars in the heat stress response. J. Exp. Bot. 60, 3891–3908. doi: 10.1093/jxb/erp234 19628571 PMC2736902

[B19] Fu C. Liu X. X. Yang W. W. Zhao C. M. Liu J. (2016). Enhanced salt tolerance in tomato plants constitutively expressing heat-shock protein in the endoplasmic reticulum. Genet. Mol. Res. 15 (2). doi: 10.4238/gmr.15028301 27421016

[B20] González-Schain N. Dreni L. Lawas L. M. F. Galbiati M. Colombo L. Heuer S.. (2016). Genome-wide transcriptome analysis during anthesis reveals new insights into the molecular basis of heat stress responses in tolerant and sensitive rice varieties. Plant Cell Physiol. 57 (1), 57–68. doi: 10.1093/pcp/pcv174 26561535

[B21] Guan Q. M. Yue X. L. Zeng H. T. Zhu J. H. (2014). The protein phosphatase RCF2 and its interacting partner NAC019 are critical for heat stress-responsive gene regulation and thermotolerance in *Arabidopsis* . Plant Cell 26 (1), 438–453. doi: 10.1105/tpc.113.118927 24415771 PMC3963588

[B22] Guo W. W. Zhang J. X. Zhang N. Xin M. M. Peng H. R. Hu Z. R.. (2015). The wheat NAC transcription factor TaNAC2L is regulated at the transcriptional and post-translational levels and promotes heat stress tolerance in transgenic arabidopsis. PLoS One 10, 1–11. doi: 10.1371/journal.pone.0135667 PMC4549282 26305210

[B23] He G. H. Xu J. Y. Wang Y. X. Liu J. M. Li P. S. Chen M.. (2016). Drought-responsive WRKY transcription factor genes TaWRKY_1_ and TaWRKY_33_ from wheat confer drought and/or heat resistance in Arabidopsis. BMC Plant Biol. 16, 116. doi: 10.1186/s12870-016-0806-4 27215938 PMC4877946

[B24] Higashi Y. Okazaki Y. Myouga F. Shinozaki K. Saito K. (2015). Landscape of the lipidome and transcriptome under heat stress in Arabidopsis thaliana. Sci. Rep. 5 (1), 1–11. doi: 10.1038/srep10533 PMC4444972 26013835

[B25] Hwang S. G. Lee C. Y. Tseng C. S. (2018). Heterologous expression of rice *9-cis-epoxycarotenoid dioxygenase 4 (OsNCED4)* in *Arabidopsis* confers sugar oversensitivity and drought tolerance. Bot. Stud. 59 (1), 1–12. doi: 10.1186/s40529-018-0219-9 29335785 PMC5768580

[B26] Ibanez C. Poeschl Y. Peterson T. Bellstadt J. Denk K. Gogol-Doring A.. (2017). Ambient temperature and genotype differentially affect developmental and phenotypic plasticity in *Arabidopsis* thaliana. BMC Plant Biol. 17, 114. doi: 10.1186/s12870-017-1068-5 28683779 PMC5501000

[B27] Ji X. M. Dong B. D. Shiran B. Talbot M. J. Edlington J. E. Hughes T.. (2011). Control of abscisic acid catabolism and abscisic acid homeostasis is important for reproductive stage stress tolerance in cereals. Plant Physiol. 156 (2), 647–662. doi: 10.1104/pp.111.176164 21502188 PMC3177265

[B28] Jia X. Y. Xu C. Y. Jing R. L. Li R. Z. Mao X. G. Wang J. P.. (2008). Molecular cloning and characterization of wheat calreticulin (CRT) gene involved in drought-stressed responses. J. Exp. Bot. 59 (4), 739–751. doi: 10.1093/jxb/erm369 18349049

[B29] Jiang H. Du J. Mao L. Li Y. Yue Y. Lu J. (2020). Summary of transcription factors in response to HT stress in plants. Mol. Plant Breed. 18 (10), 3251–3258. doi: 10.13271/j.mpb.018.003251

[B30] Jiang J. Liu X. Liu C. Liu G. Li S. Wang L. (2017). Integrating omics and alternative splicing reveals insights into grape response to HT. Plant Physiol. 173 (2), 1502–1518. doi: 10.1104/pp.16.01305 28049741 PMC5291026

[B31] Jin J. Yang L. Fan D. Liu X. Hao Q. (2020). Comparative transcriptome analysis uncovers different heat stress responses in heat-resistant and heat-sensitive jujube cultivars. PLoS One 15 (9), e0235763. doi: 10.1371/journal.pone.0235763 32956359 PMC7505471

[B32] Jung K. H. Ko H. J. Nguyen M. Kim S. R. Ronald P. An G. (2012). Genome-wide identification and analysis of early heat stress responsive genes in rice. J. Plant Biol. 55, 458–468. doi: 10.1007/s12374-012-0271-z

[B33] Kana T. Kazumasa M. Takuya Y. Kohei Y. Genki C. Shintaro K.. (2018). Thermo-responsive allele of sucrose synthase 3 (Sus3) provides high-temperature tolerance during the ripening stage in rice (Oryza sativa l.). Breed. Sci. 68 (3), 336–342. doi: 10.1270/jsbbs.18007 30100800 PMC6081304

[B34] Keller M. Bokszczanin K. Bostan H. Bovy A. G. Simm S. (2018). The coupling of transcriptome and proteome adaptation during development and heat stress response of tomato pollen. BMC Genomics 19 (1), 447. doi: 10.1186/s12864-018-4824-5 29884134 PMC5994098

[B35] Kim S. A. Ahn S. Y. Yun H. K. (2018). Selection of differentially expressed genes using the transcriptome analysis of ripening grape berries in response to HT. J. Agric. Sci-Sri. Lanka. 13, 15–30. doi: 10.4038/jas.v13i1.8297

[B36] Kumar S. Gupta D. Nayyar H. (2012). Comparative response of maize and rice genotypes to heat stress: status of oxidative stress and antioxidants. Acta Physiologiae Plantarum 34 (1), 75–86. doi: 10.1007/s11738-011-0806-9

[B37] Langfelder P. Horvath S. (2008). WGCNA: An R package for weighted correlation network analysis. BMC Bioinform. 9, 559–571. doi: 10.1186/1471-2105-9-559 PMC2631488 19114008

[B38] Larkindale J. Huang B. (2004). Changes of lipid composition and saturation level in leaves and roots for heat-stressed and heat-acclimated creeping bentgrass (*Agrostis stolonifera*). Environ. Exp. Bot. 51 (1), 57–67. doi: 10.1016/S0098-8472(03)00060-1

[B39] Lecourieux F. Kappel C. Pieri P. Charon J. Pillet J. Hilbert G.. (2017). Dissecting the biochemical and transcriptomic effects of a locally applied heat treatment on developing Cabernet Sauvignon grape berries. Front. Plant Sci. 8. doi: 10.3389/fpls.2017.00053 PMC5281624 28197155

[B40] Lenka S. K. Muthusamy S. K. Chinnusamy V. Bansal K. C. (2018). Ectopic expression of rice PYL3 enhances cold and drought tolerance in *Arabidopsis thaliana* . Mol. Biotechnol. 60 (5), 350–361. doi: 10.1007/s12033-018-0076-5 29574592

[B41] Li S. J. Fu Q. T. Chen L. G. Huang W. D. Yu D. Q. (2011). Arabidopsis thaliana WRKY25, WRKY26, and WRKY33 coordinate induction of plant thermotolerance. Planta 233, 1237–1252. doi: 10.1007/s00425-011-1375-2 21336597

[B42] Li C. Li C. Wang B. B. Zhang R. Q. Fu K. Y. Gale W. L.. (2018). Programmed cell death in wheat (*Triticum aestivum* L.) endosperm cells is affected by drought stress. Protoplasma 255 (4), 1039–1052. doi: 10.1007/s00709-018-1203-7 29380071

[B43] Li T. Xu X. Li Y. Wang H. M. Li Z. L. Li Z. X. (2015). Comparative transcriptome analysis reveals differential transcription in heat-susceptible and heat-tolerant pepper (*Capsicum annum* L.) cultivars under heat stress. J. Plant Biol. 58 (6), 411–424. doi: 10.1007/s12374-015-0423-z

[B44] Liu M. Ju Y. L. Min Z. Fang Y. L. Meng J. F. (2020). Transcriptome analysis of grape leaves reveals insights into response to heat acclimation. Scientia Hortic. 272, 109554. doi: 10.1016/j.scienta.2020.109554

[B45] Liu B. L. Kong L. S. Zhang Y. Liao Y. C. (2021). Gene and metabolite integration analysis through transcriptome and metabolome brings new insight into heat stress tolerance in potato (*Solanum tuberosum* L.). Plants 10 (1), 103. doi: 10.3390/plants10010103 33419030 PMC7825342

[B46] Liu G. T. Wang J. F. Cramer G. Dai Z. W. Duan W. Xu H. G.. (2012). Transcriptomic analysis of grape (*Vitis vinifera* L.) leaves during and after recovery from heat stress. BMC Plant Biol. 12 (1), 174. doi: 10.1186/1471-2229-12-174 23016701 PMC3497578

[B47] Liu H. Wang J. Sun H. M. Han X. B. Du B. H. (2020). Transcriptome profiles reveal the growth-promoting mechanisms of Paenibacillus polymyxa *YC0136* on tobacco (*Nicotiana tabacum* L.). Front. Microbiol. 11. doi: 10.3389/fmicb.2020.584174 PMC7546199 33101258

[B48] Liu S. L. Yang R. J. Pan Y. Z. Ding J. J. He Y. Wang L. (2013). Effects of exogenous nitric oxide on lipid peroxidation and ATPase activity in plasma membrane and photosynthetic characteristics of catharanthus roseus under cadmium stress. J. Agro-Environment Sci. 32 (12), 2360–2368. doi: 10.11564/jaes.2013.12.008

[B49] Liu G. Zha Z. P. Cai H. Y. Qin D. D. Jiao C. H. (2020). Dynamic transcriptome analysis of anther response to heat stress during anthesis in thermotolerant rice (*Oryza sativa* L.). Int. J. Mol. Sci. 21 (3), 1155. doi: 10.3390/ijms21031155 32050518 PMC7037497

[B50] Liu J. Zhao F. L. Guo Y. Fan X. C. Wang Y. J. Wen Y. Q. (2019). The ABA receptor-like gene *VyPYL9* from drought-resistance wild grapevine confers drought tolerance and ABA hypersensitivity in *Arabidopsis* . Plant Cell Tissue Organ Cult. 138 (3), 543–558. doi: 10.1007/s11240-019-01650-2

[B51] Londo J. P. Kovaleski A. P. Lillis J. A. (2018). Divergence in the transcriptional landscape between low temperature and freeze shock in cultivated grapevine (Vitis vinifera). Hortic. Res. 5, 10. doi: 10.1038/s41438-018-0020-7 29507734 PMC5830407

[B52] Luo H. B. Ling M. Xi H. F. Duan W. Li S. H. Wayne L.. (2011). Photosynthetic responses to heat treatments at different temperatures and following recovery in grapevine (*vitis amurensis* L.) leaves. PLoS One 6 (8), e23033. doi: 10.1371/journal.pone.0023033 21887227 PMC3162573

[B53] Ma C. Burd S. Lers A. (2015). miR408 is involved in abiotic stress responses in arabidopsis. Plant J. 84 (1), 169–187. doi: 10.1111/tpj.12999 26312768

[B54] Manabe Y. Bressan R. A. Wang T. Li F. Koiwa H. Sokolchik I.. (2007). The Arabidopsis kinase-associated protein phosphatase regulates adaptation to Na^+^ stress. Plant Physiol. 146 (2), 612–622. doi: 10.1104/pp.107.109009 18162596 PMC2245828

[B55] Meng Z. Zhao Y. Liu L. Du X. (2021). Genome-wide characterization of the PDI gene family in medicago truncatula and their roles in response to endoplasmic reticulum stress. Genome 64 (6), 599–614. doi: 10.1139/gen-2020-0064 33306442

[B56] Moreno A. A. (2021). The endoplasmic reticulum role in the plant response to abiotic stress. Front. Plant Sci. 12. doi: 10.3389/fpls.2021.755447 PMC8637532 34868142

[B57] Nadarajah K. K. (2020). ROS homeostasis in abiotic stress tolerance in plants. Int. J. Mol. Sci. 21 (15), 5208. doi: 10.3390/ijms21155208 32717820 PMC7432042

[B58] Ng D. W. K. Abeysinghe J. K. Kamali M. (2018). Regulating the regulators: the control of transcription factors in plant defense signaling. Int. J. Mol. Sci. 19, 3737. doi: 10.3390/ijms19123737 30477211 PMC6321093

[B59] Nishizawa A. Yabuta Y. Yoshida E. Maruta T. Yoshimura K. Shigeoka S. (2006). Arabidopsis heat shock transcription factor *A2* as a key regulator in response to several types of environmental stress. Plant J. 48, 535–547. doi: 10.1111/j.1365-313X.2006.02889.x 17059409

[B60] Ozgur R. Turkan I. Uzilday B. Sekmen A. H. (2014). Endoplasmic reticulum stress triggers ROS signalling, changes the redox state, and regulates the antioxidant defence of arabidopsis thaliana. J. Exp. Bot. 65, 1377–1390. doi: 10.1093/jxb/eru034 24558072 PMC3969530

[B61] Park C. J. Seo Y. S. (2015). Heat shock proteins: a review of the molecular chaperones for plant immunity. Plant Pathol. J. 31 (4), 323. doi: 10.5423/PPJ.RW.08.2015.0150 26676169 PMC4677741

[B62] Parrotta L. Faleri C. Cresti M. Cai G. (2016). Heat stress affects the cytoskeleton and the delivery of sucrose synthase in tobacco pollen tubes. Planta 243 (1), 43–63. doi: 10.1007/s00425-015-2394-1 26335855

[B63] Payne C. M. Knott B. C. Mayes H. B. Hansson H. Himmel M. E. Sandgren M.. (2015). Fungal cellulases. Chem. Rev. 115 (3), 1308–1448. doi: 10.1021/cr500351c 25629559

[B64] Pizzio G. A. Rodriguez L. Antoni R. Gonzalez-Guzman M. Yunta C. Merilo E.. (2013). The PYL4 A194T mutant uncovers a key role of PYR1-LIKE4/PROTEIN PHOSPHATASE 2CA interaction for abscisic acid signaling and plant drought resistance. Plant Physiol. 163 (1), 441–455. doi: 10.1104/pp.113.224162 23864556 PMC3762663

[B65] Qi Y. C. Wang H. J. Zou Y. Liu C. Liu Y. Q. Wang Y.. (2010). Over-expression of mitochondrial heat shock protein 70 suppresses programmed cell death in rice. FEBS Lett. 585 (1), 231–239. doi: 10.1016/j.febslet.2010.11.051 21130768

[B66] Rahmati I. M. Brown E. Weigand C. Tillett R. L. Schlauch K. A. Miller G.. (2018). A comparison of heat-stress transcriptome changes between wild-type Arabidopsis pollen and a heat-sensitive mutant harboring a knockout of cyclic nucleotide-gated cation channel 16 (*cngc16*). BMC Genomics 19 (1), 1–19. doi: 10.1186/s12864-018-4930-4 30041596 PMC6057101

[B67] Reyes-Impellizzeri S. Moreno A. A. (2021). The endoplasmic reticulum role in the plant response to abiotic stress. Front. Plant Sci. 12. doi: 10.3389/fpls.2021.755447 PMC8637532 34868142

[B68] Rienth M. Torregrosa L. Luchaire N. Chatbanyong R. Lecourieux D. Kelly M. T.. (2014). Day and night heat stress trigger different transcriptomic responses in green and ripening grapevine (*Vitis vinifera*) fruit. BMC Plant Biol. 14 (1), 1–18. doi: 10.1186/1471-2229-14-108 PMC4030582 24774299

[B69] Ruan Y. L. (2014). Sucrose metabolism: gateway to diverse carbon use and sugar signaling. Annu. Rev. Plant Biol. 65 (1), 33–67. doi: 10.1146/annurev-arplant-050213-040251 24579990

[B70] Ruelland E. Zachowski A. (2010). How plants sense temperature. Environ. Exp. Bot. 69 (3), 225–232. doi: 10.1016/j.envexpbot.2010.05.011

[B71] Salehin M. Li B. Tang M. Katz E. Song L. Ecker J. R.. (2019). Auxin-sensitive Aux/IAA proteins mediate drought tolerance in *Arabidopsis* by regulating glucosinolate levels. Nat. Commun. 10 (1), 4021. doi: 10.1038/s41467-019-12002-1 31492889 PMC6731224

[B72] Schneider J. R. Caverzan A. Chavarria G. (2019). Water deficit stress, ROS involvement, and plant performance. Arch. Agron. Soil Sci. 65 (8), 1160–1181. doi: 10.1080/03650340.2018.1556789

[B73] Schultz H. R. Jones G. V. (2010). Climate induced historic and future changes in viticulture. J. Wine Res. 21 (2-3), 137–145. doi: 10.1080/09571264.2010.530098

[B74] Shani E. Salehin M. Zhang Y. Q. Sanchez S. E. Doherty C. Wang R. H.. (2016). Plant stress tolerance requires Auxin-sensitive Aux/IAA transcriptional repressors. Curr. Biol. 27 (3), 437–444. doi: 10.1016/j.cub.2016.12.016 PMC5296222 28111153

[B75] Singh G. Sarkar N. K. Grover A. (2018). Mapping of domains of heat stress transcription factor *OsHsfA6a* responsible for its transacti vation activity. Plant Sci. 274, 80–90. doi: 10.1016/j.plantsci.2018.05.010 30080644

[B76] Song C. Chung W. S. Lim C. O. (2016). Overexpression of heat shock factor gene *HsfA3* Increases galactinol levels and oxidative stress tolerance in arabidopsis. Mol. Cells 39, 477–483. doi: 10.14348/molcells.2016.0027 27109422 PMC4916399

[B77] Suzuki N. Bassil E. Hamilton J. S. Inupakutika M. A. Zandalinas S. I. Tripathy D.. (2016). ABA is required for plant acclimation to a combination of salt and heat stress. PLoS One 11 (1), e0147625. doi: 10.1371/journal.pone.0147625 26824246 PMC4733103

[B78] Tang R. M. Gupta S. K. Niu S. Y. Li X. Q. Yang Q. Chen G. S.. (2020). Transcriptome analysis of heat stress response genes in potato leaves. Mol. Biol. Rep. 47 (6), 4311–4321. doi: 10.1007/s11033-020-05485-5 32488578

[B79] Verma E. Sharma B. Singal H. R. Munjal R. (2018). Purification of sucrose synthase from thermotolerant wheat grains and its characterization. J. Environ. Biol. 39 (4), 459–466. doi: 10.22438/jeb/39/4/MRN-503

[B80] Wahid A. Gelani S. Ashraf M. Foolad M. R. (2007). Heat tolerance in plants: An overview. Environ. Exp. Bot. 61 (3), 199–223. doi: 10.1016/j.envexpbot.2007.05.011

[B81] Wai A. H. Waseem M. Khan A.B.M.M.M. Nath U. K. Lee D. J. Kim S. T.. (2020). Genome-wide identification and expression profiling of the PDI gene family reveals their probable involvement in abiotic stress tolerance in tomato (Solanum lycopersicum l.). Genes (Basel) 12 (1), 23. doi: 10.3390/genes12010023 33375673 PMC7824348

[B82] Wang L. J. Fan L. Loescher W. Duan W. Liu G. J. Cheng J. S.. (2010). Salicylic acid alleviates decreases in photosynthesis under heat stress and accelerates recovery in grapevine leaves. BMC Plant Biol. 10, 34. doi: 10.1186/1471-2229-10-34 20178597 PMC2848757

[B83] Wang L. J. Li S. H. (2006). Salicylic acid-induced heat or cold tolerance in relation to Ca^2+^ homeostasis and antioxidant systems in young grape plants. Plant Sci. 170 (4), 685–694. doi: 10.1016/j.plantsci.2005.09.005

[B84] Wang W. L. Lin Y. H. Teng F. Ji D. H. Xu Y. Chen C. S.. (2018). Comparative transcriptome analysis between heat-tolerant and sensitive Pyropia haitanensis strains in response to HT stress. Algal Res. 29, 104–112. doi: 10.1016/j.algal.2017.11.026

[B85] Wang L. J. Loescher W. Duan W. Li W. D. Yang S. H. Li S. H. (2009). Heat acclimation induced acquired heat tolerance and cross adaptation in different grape cultivars: relationships to photosynthetic energy partitioning. Funct. Plant Biol. 36, 516–526. doi: 10.1071/FP09008 32688666

[B86] Wang H. Niu H. Zhai Y. Lu M. (2017). Characterization of B*iP* genes from pepper (C*apsicum annuum* l.) and the role of C*aBiP1* in response to endoplasmic reticulum and multiple abiotic stresses. Front. Plant Sci. 8. doi: 10.3389/fpls.2017.01122 PMC5487487 28702041

[B87] Wang L. Xiang L. Hong J. Xie Z. Li B. (2019). Genome-wide analysis of bHLH transcription factor family reveals their involvement in biotic and abiotic stress responses in wheat (*Triticum aestivum* L.). 3 Biotech. 9 (6), 236. doi: 10.1007/s13205-019-1742-4 PMC6536565 31139551

[B88] Wang Y. L. Zhang Y. K. Shi Q. H. Chen H. Z. Xiang J. Hu G. H.. (2020). Decrement of sugar consumption in rice young panicle under HT aggravates spikelet number reduction. Rice Sci. 27 (01), 44–55. doi: 10.1016/j.rsci.2019.12.005

[B89] Wu J. Y. Liu L. Y. Xu G. X. Jiang J. F. Lian W. J. Zhou H.. (2021). Grape planting situation and regional spatial analysis in Xinjiang, China. IOP Conf. Ser.: Earth Environ. Sci. 705, 12028. doi: 10.1088/1755-1315/705/1/012028

[B90] Xiang Y. Lu Y. H. Song M. Wang Y. Xu W. Q. Wu L. T.. (2015). Overexpression of a triticum aestivum calreticulin gene (TaCRT1) improves salinity tolerance in tobacco. PloS One 10 (10), e0140591. doi: 10.1371/journal.pone.0140591 26469859 PMC4607401

[B91] Zhang A. Y. Yang X. Lu J. Song F. Y. Sun J. H. Wang C.. (2021). OsIAA20, an Aux/IAA protein, mediates abiotic stress tolerance in rice through an ABA pathway. Plant Sci. 308, 110903. doi: 10.1016/j.plantsci.2022.111403 34034863

[B92] Zhao Y. Tian X. Wang F. Zhang L. Xin M. Hu Z.. (2017). Characterization of wheat MYB genes responsive to high temperatures. BMC Plant Biol. 17 (1), 208. doi: 10.1186/s12870-017-1158-4 29157199 PMC5696766

[B93] Zhou H. Wang Y. F. Zhang Y. J. Xiao Y. H. Liu X. Deng H. B.. (2022). Comparative analysis of heat-tolerant and heat-susceptible rice highlights the role of *OsNCED1* gene in heat stress tolerance. Plants (Basel) 11 (8), 1062. doi: 10.3390/plants11081062 35448790 PMC9026844

[B94] Zhou Z. P. Yuan Y. Z. Zhou W. Zhang C. F. (2016). Effects of exogenously supplied sucrose on OsSUTs and OsSPSs transcript abundances and rice root ammonium assimilation. Acta Physiol. Plant 38, 274. doi: 10.1007/s11738-016-2285-5

[B95] Zhu J. K. (2016). Abiotic stress signaling and responses in plants. Cell 167, 313–324. doi: 10.1016/j.cell.2016.08.029 27716505 PMC5104190

