# Supplementary figures and images for: Transcriptomic and physiological analyses reveal different grape varieties response to high temperature stress
Source: Front Plant Sci. 2024 Mar 8;15:1313832. doi: 10.3389/fpls.2024.1313832 (PMC10957553; doi:10.3389/fpls.2024.1313832)

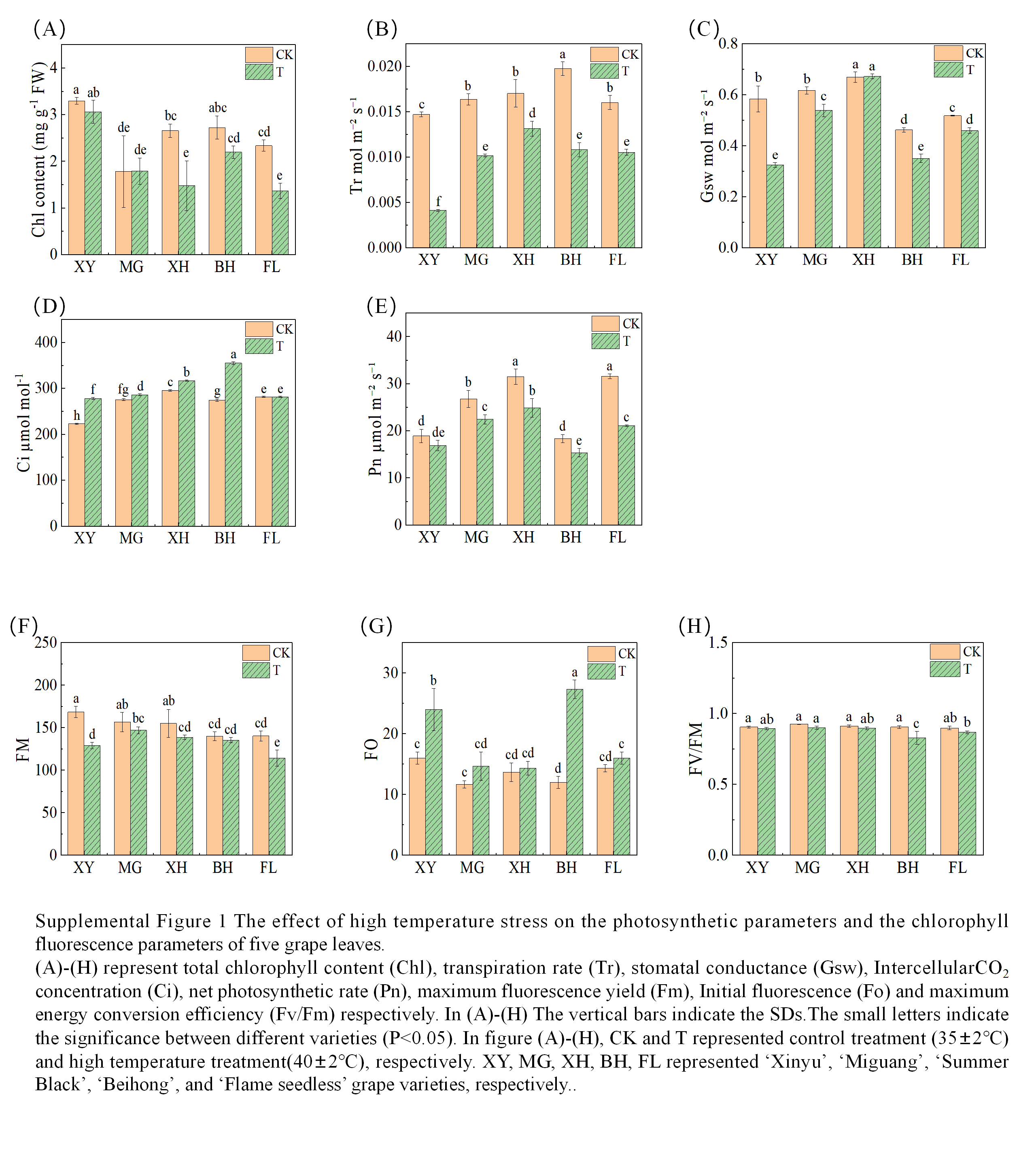

Supplement: Supplementary file 1 [file DataSheet_1.zip › supplementary materials/Figure S1.png]

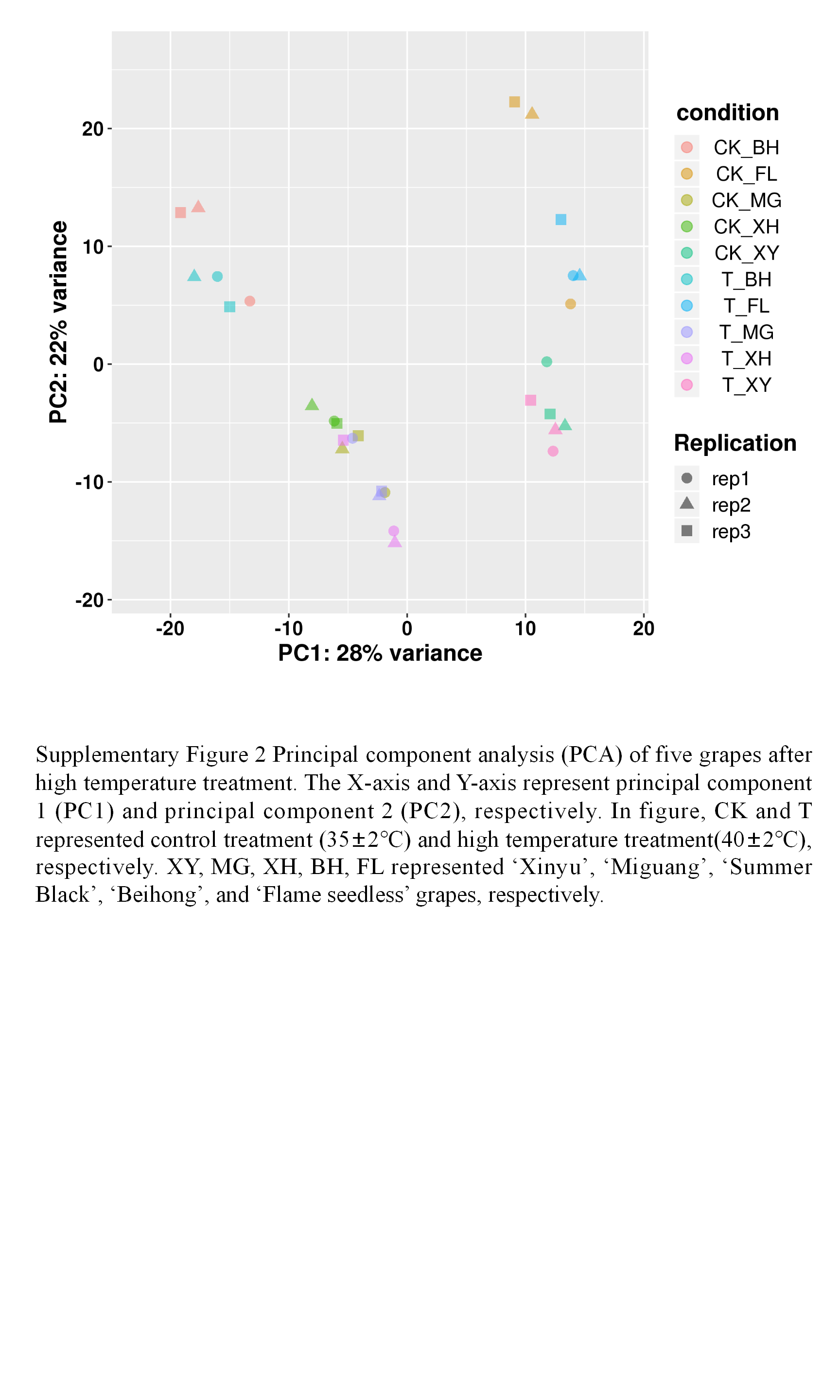

Supplement: Supplementary file 1 [file DataSheet_1.zip › supplementary materials/Figure S2.png]

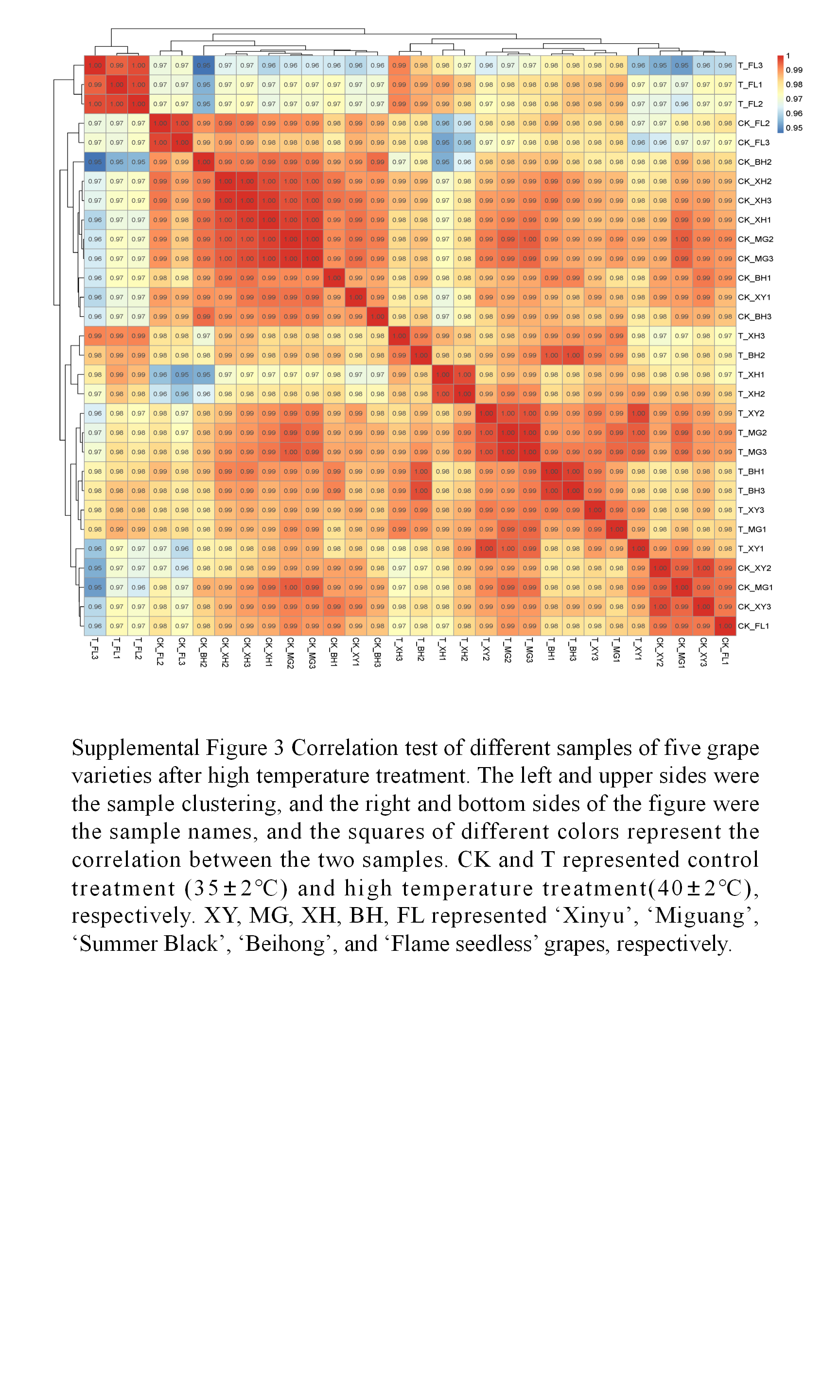

Supplement: Supplementary file 1 [file DataSheet_1.zip › supplementary materials/Figure S3.png]

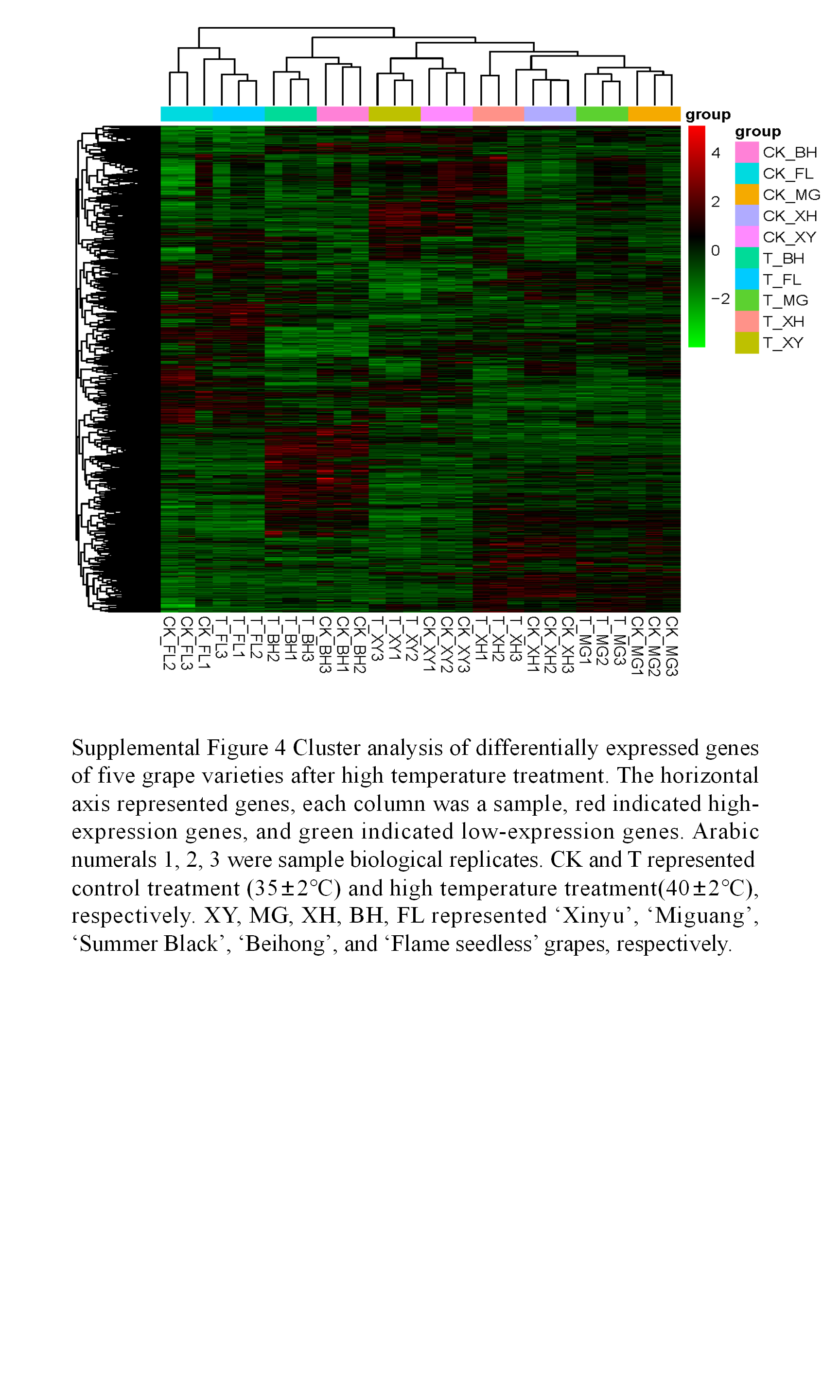

Supplement: Supplementary file 1 [file DataSheet_1.zip › supplementary materials/Figure S4.png]

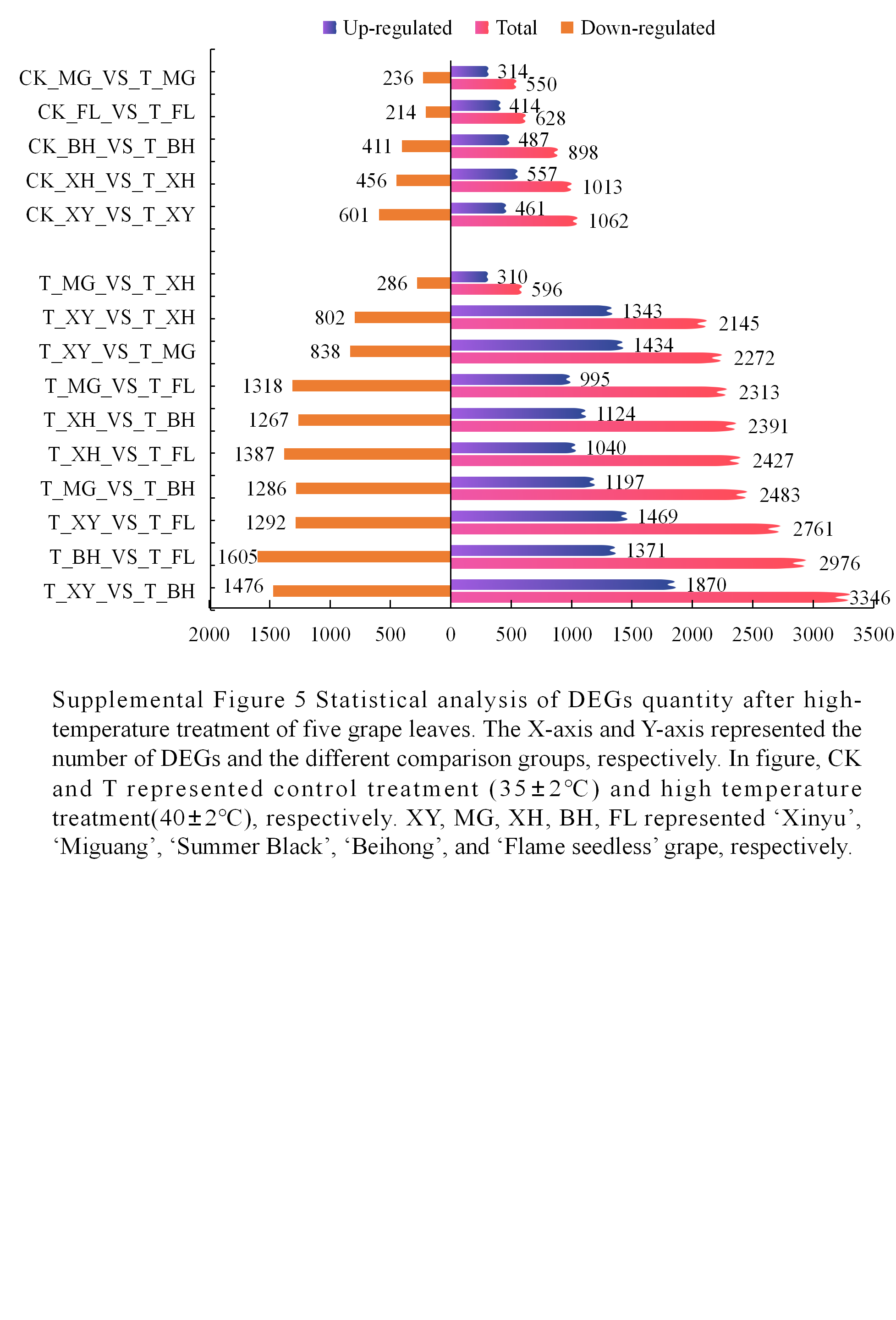

Supplement: Supplementary file 1 [file DataSheet_1.zip › supplementary materials/Figure S5.png]

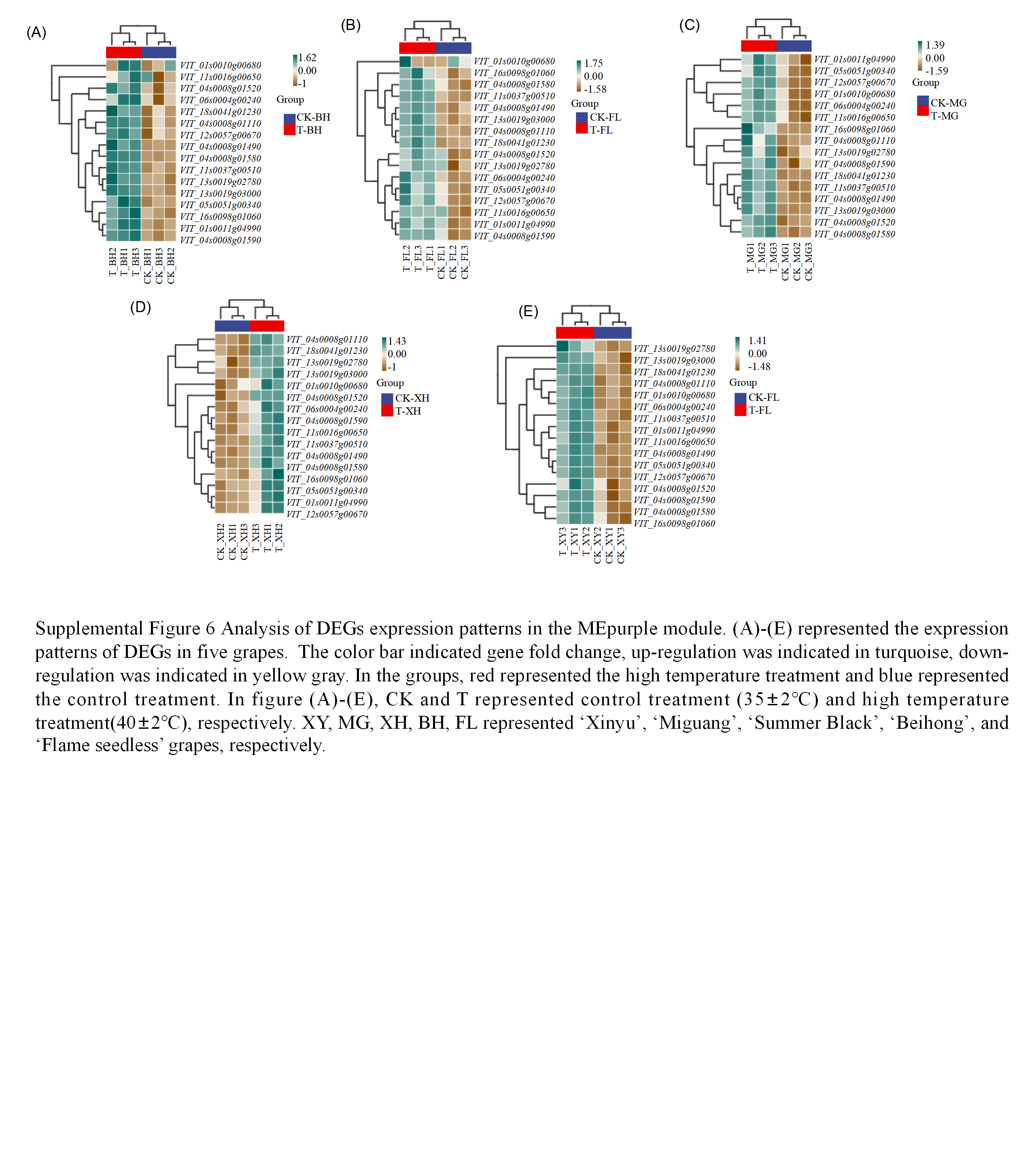

Supplement: Supplementary file 1 [file DataSheet_1.zip › supplementary materials/Figure S6.png]

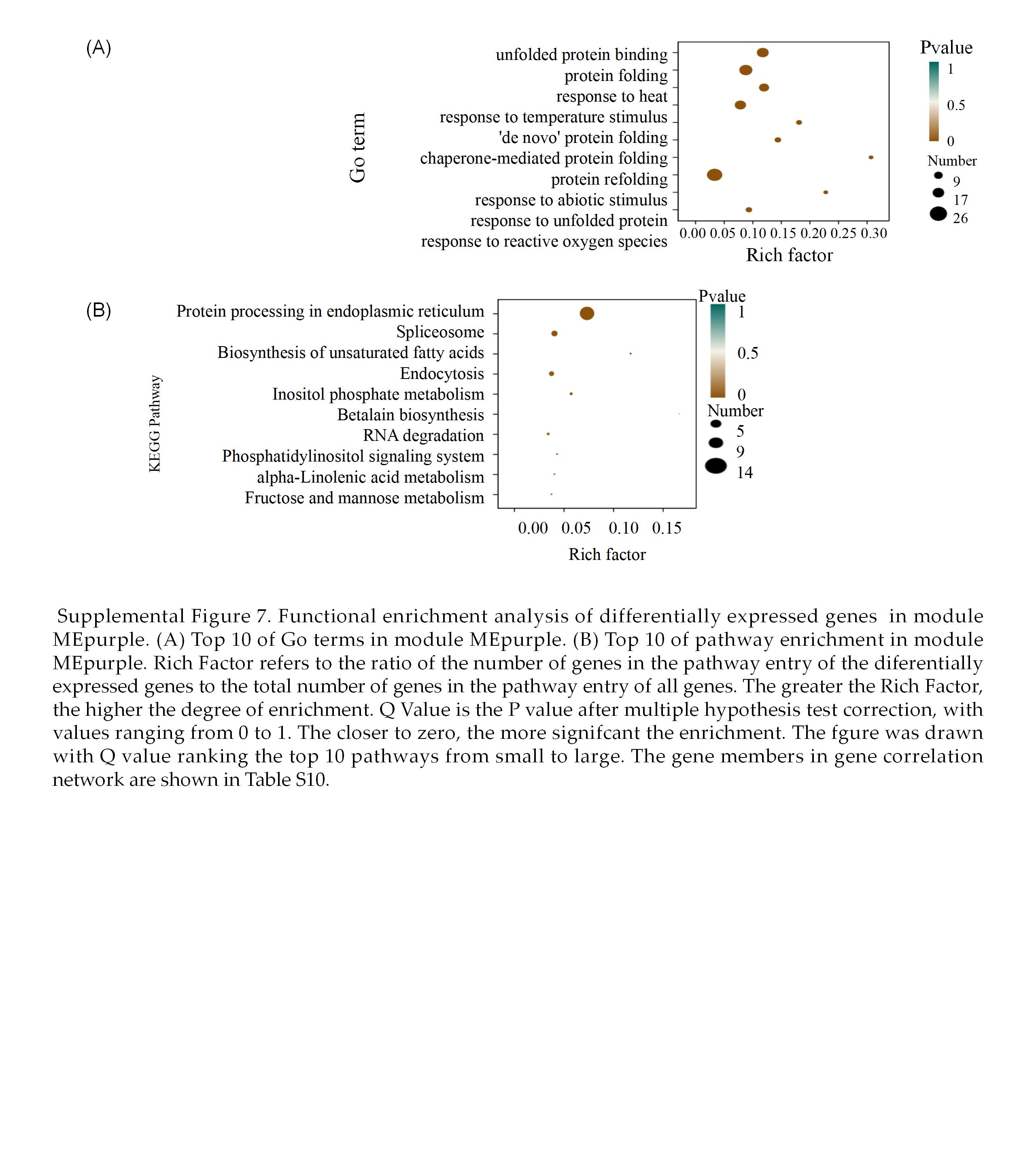

Supplement: Supplementary file 1 [file DataSheet_1.zip › supplementary materials/Figure S7.jpg]

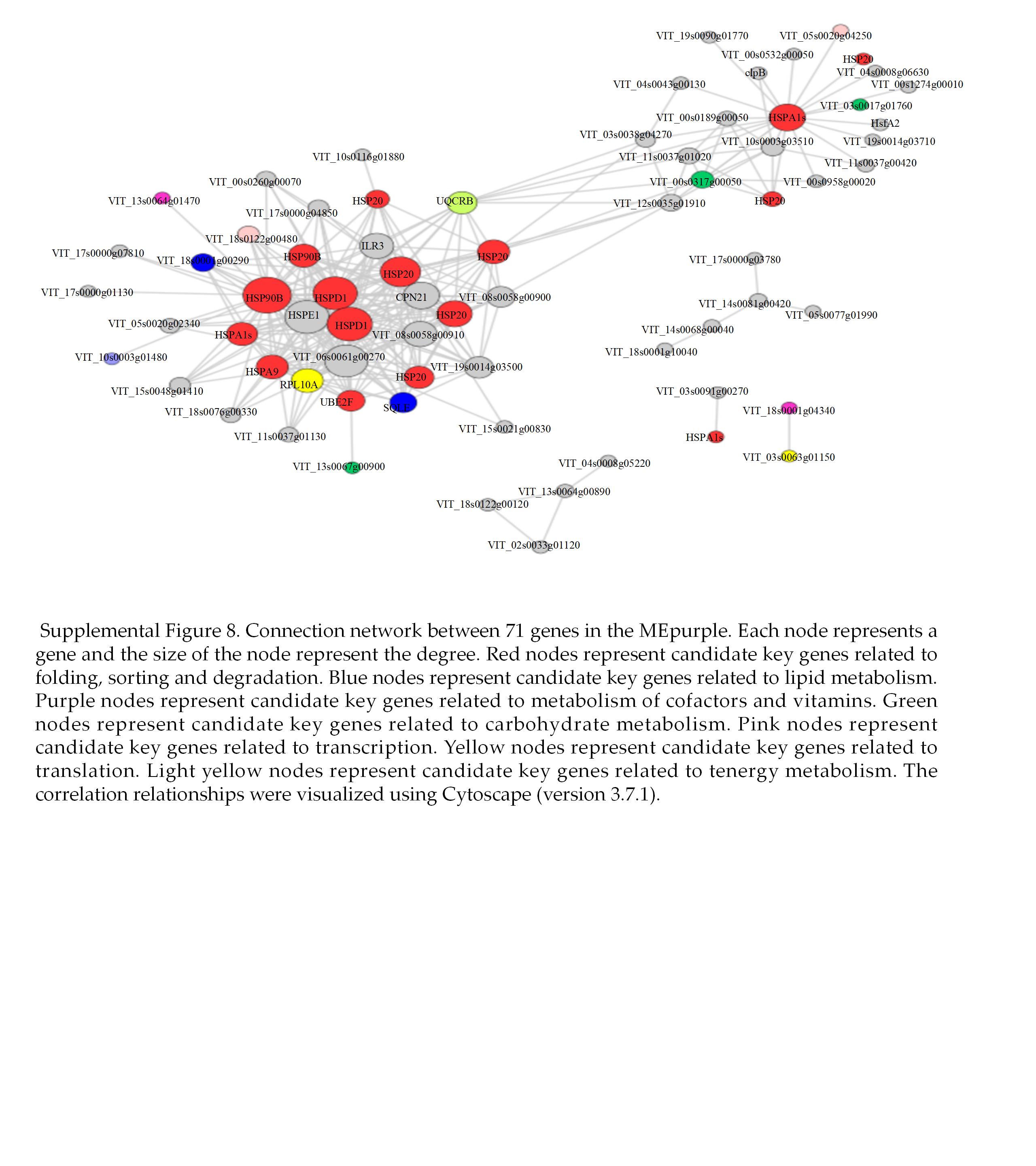

Supplement: Supplementary file 1 [file DataSheet_1.zip › supplementary materials/Frgure S8.jpg]
